# The housing environment of participants in the Avon Longitudinal Study of Parents and Children (ALSPAC): a resource for studies of influences on health

**DOI:** 10.12688/wellcomeopenres.17996.1

**Published:** 2022-09-12

**Authors:** Genette Ellis, Yasmin Iles-Caven, Kate Northstone, Jean Golding

**Affiliations:** 1 Centre for Academic Child Health, Population Health Sciences, Bristol Medical School, University of Bristol, Oakfield House, Oakfield Grove, Bristol, BS8 2BN, UK; 2 Population Health Sciences, Bristol Medical School, University of Bristol, Oakfield House, Oakfield Grove, Bristol, BS8 2BN, UK

**Keywords:** ALSPAC, housing, environment, well-being, physical health, childhood, longitudinal cohort

## Abstract

**Background:**Early life environmental health exposures related to housing can have a significant impact on an individual’s physical and mental health and physical development. Housing exposures can fall into two main areas – a representation of social circumstances and physical conditions.

**Methods:**During pregnancy and post-delivery, self-completion questionnaires concerning the housing environment were administered until the study offspring were aged 18 to the ALSPAC (Avon Longitudinal Study of Parents & Children) study mothers and their partners. Data collected included types of housing, housing tenure, numbers of rooms in the home, whether shared or sole use of kitchen and indoor flushing toilet, household moves, Council Tax band, difficulties in affording rent/mortgage and becoming homeless. Here we also describe the historic development of housing in the Bristol and surrounding Avon areas.

**Results:** Data collected included the tenure (e.g., owned/rented) of the home, its size (indexed by the number of rooms), the presence of over-crowding (measured by the number of residents per room), presence of amenities, and frequency of household moves. This information was collected on over 13,000 women during pregnancy >8000 at age 10 and >4000 at 18 years. Council Tax bands were asked at 10 and 18 years.

**Conclusions:** This is the first of two Data Notes on the housing type and housing circumstances of the families enrolled in ALSPAC. A second Data Note will detail their internal housing conditions. The data provides an excellent resource for researchers when considering the influences of housing on physical and mental health and development.

## Introduction

Housing quality and housing instability can have significant impacts on physical and psychological health, including increased instances of depression and anxiety in women ( Suglia *et al.*, 2011); morbidity in infancy (e.g. Baker *et al.*, 1998); asthma and allergies in childhood (e.g. Choi *et al.*, 2017); and general ill-health (e.g. Marsh *et al.*, 2000). Therefore, housing type may be an important consideration when looking at the prevalence of morbidity in parents and their offspring. In this paper we describe the data collected from families enrolled in the Avon Longitudinal Study of Parents and Children (ALSPAC), centred on the city of Bristol and surrounding areas in South-West England, regarding their housing type and circumstances during the first 18 years of life. The study provides an excellent resource for researchers when considering health and/or developmental outcomes for the study children and/or their parents.

### Historical background and the manmade environment

There is evidence that Bristol and the surrounding areas of the former county of Avon have been inhabited by humans since the Stone Age, with various archaeological sites such as Stanton Drew stone circle ( David *et al.*, 2004) and barrows (e.g. the bowl barrow at Southmead), iron age hill forts (e.g. in Henbury), Clifton Hill and Kings Weston Hill camps and the Druid Stoke burial chamber (in Stoke Bishop) ( Ancient & Scheduled Monuments, 2022). The ancient settlement of *Brycgstow* (Old English: Place of the Bridge) grew up around the confluence of two rivers, the Frome and the Avon (which gave sea access via the River Severn at Avonmouth) before 1000 A.D. By 1066, the population was around 4000. The port was already important between the 11 ^th^ and 13 ^th ^centuries (in the 1240s the city port was expanded by diverting the river Frome into a deep canal (St. Augustine’s Reach). At this time, wine was the main import (from Spain, Portugal and Southwest France). Bristol exports included dyed woollen products, lead, sail cloth and rope. However, one of the exports to Dublin between the 10 ^th^ and 15 ^th^ centuries was Welsh and Northern English slaves. By the 1460s it was the country’s second port trading with Ireland, Iceland and Gascony. Around 1480, Bristol’s Society of Merchant Venturers begin sponsoring exploration of the north Atlantic. Bristol attained city status in 1542 ( Wikipedia, 2022).

As such, its city centre buildings and those in the surrounding area range in date and style from the Tudor/Elizabethan (1485-1603), Georgian (1714-1836), Victorian (1837-1901) and Edwardian (1901-1910) eras, to inter-war housing through to the contemporary, with ongoing improvements, gentrification, repurposing, and new builds.

Much of Bristol’s housing grew up around the industries that flourished in the old city including soap, potteries and glass production, tobacco, sugar and chocolate, leather, textiles, lead shot manufacturing and breweries. These influenced the layout and expansion of housing across the city ( Harvey & Press, 1988).

By far, the biggest impact on house building in the Georgian period was the trans-Atlantic slave trade. Between 1700 and 1804, 2000 Bristol ships carried at estimated 500,000 enslaved Africans to the New World. Indeed, in 1747, it was Britain’s busiest slaving port. The city also processed many of the slave-produced goods such as sugar and tobacco. The trade brought (directly or indirectly) great wealth to many merchants in the city, who moved out of the port area and up the hill to leafy Clifton where many of the elegant townhouses, crescents and villas can be found today. After Bristol’s decline as a slaving port, Clifton had one of the highest numbers of retired plantation owners in Britain ( Lewin-Turner, 2022). Many merchants with connections to the slave trade (e.g., Edward Colston, the Farr family, the Wills family) were also great philanthropists creating schools, alms houses and the Theatre Royal.

**Figure 1.**
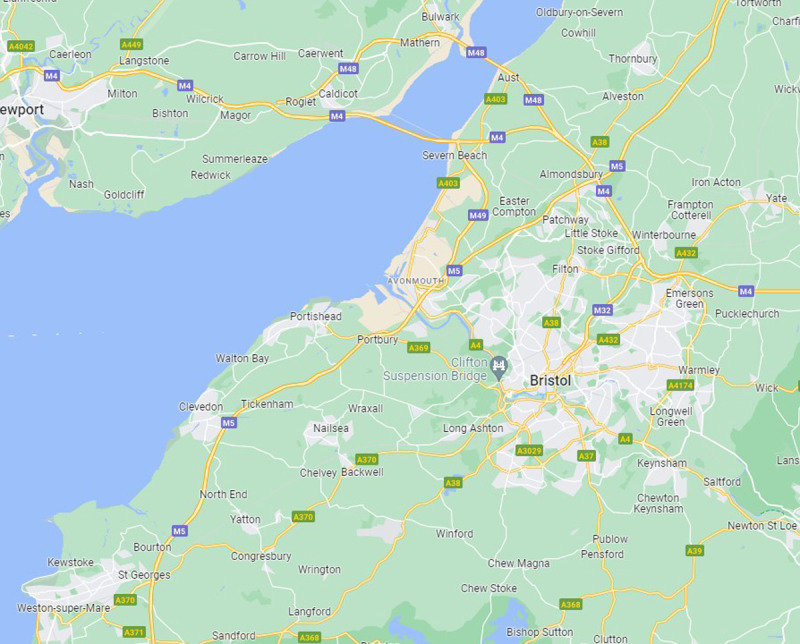
Avon area showing the main small towns and villages. Map credit: Google Maps.

Until 1949, there was extensive coal mining in the north-east of Bristol (e.g., the Kingswood and Fishponds areas) and Bedminster in the south (see Figure 1), which led to the expansion of established villages where much of the original (Victorian) housing stock remains. Currently, the city has significant aerospace, media, information technology and financial services sectors, as well as tourism and education, with two universities with a combined student population of around 60,000.

**Figure 2.**
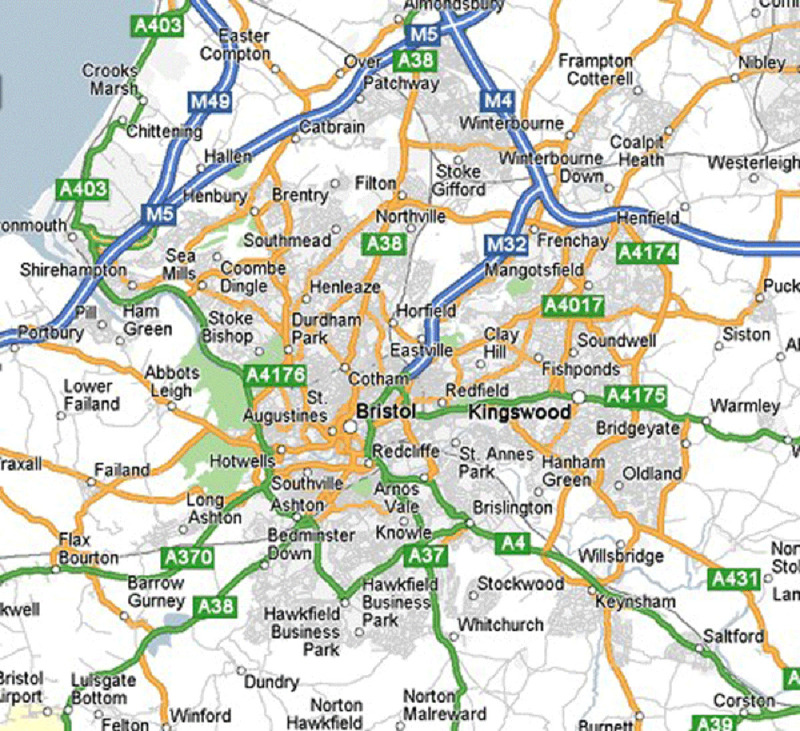
Map of Bristol and the surrounding areas indicating main arterial roads and small villages within the ALSPAC catchment area. Map credit: https://gadgets2018blog.blogspot.com/2017/10/map-of-bristol-area.html.

The city stands at the junction of the M4 and M5 motorways, with the M32 allowing direct access to the city centre ( Figure 2). Like many old British cities, the road system was not designed to cope with current traffic volumes and peak commuter time congestion. This has raised noise pollution ( BBC News England, 2015) and air quality ( Clean Air for Bristol, 2022) concerns.

### The housing stock

**Figure 3.**
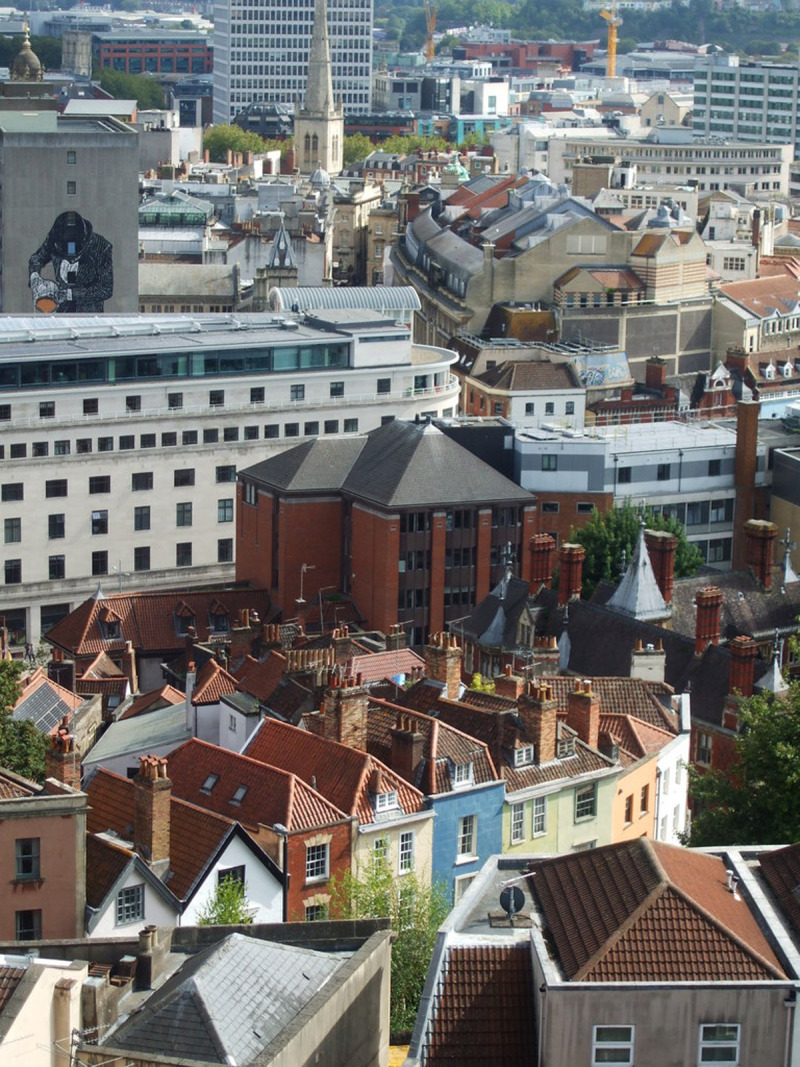
ST5873 : From the tower of St Michael on the Mount Without: Bristol city centre skyline 2019 showing modern tower blocks, ancient churches interspersed with Georgian and Victorian houses (now largely given over to offices). © Copyright Neil Owen and licensed for reuse under the Creative Commons Attribution-ShareAlike 2.0 Generic license (CC BY-SA 2.0).

**Figure 4.**
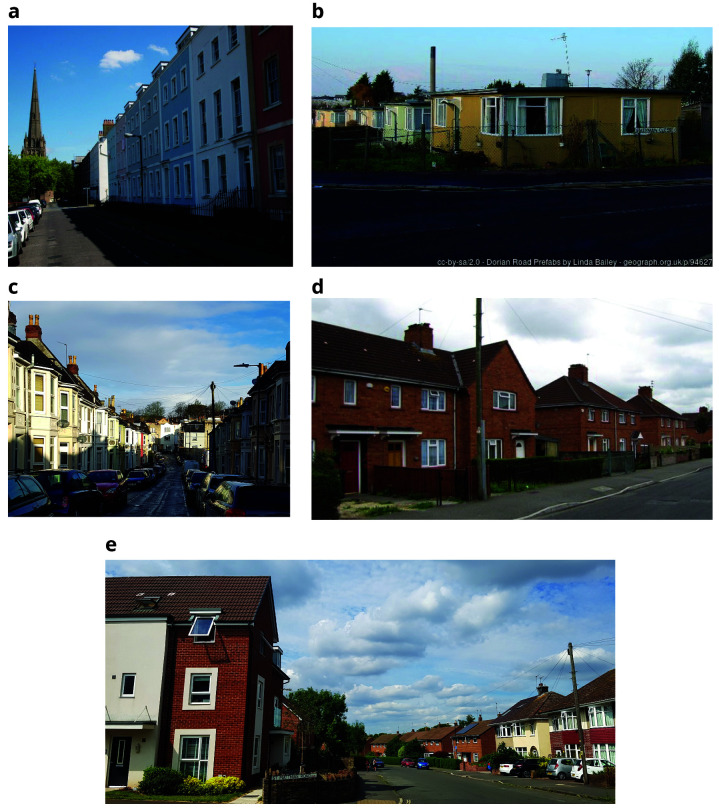
Typical Bristol housing (Top row, left: **a**. elegant Georgian terrace in central Bristol © David Hallam-Jones right: **b**. Prefab’ homes © Copyright **Linda Bailey**. Bottom row, left: **c**. Victorian terraced street; right: **d**. 1930s Council housing © Copyright Jez McNeill; **e**. Bristol suburban street showing 1930s semi-detached homes, 1950s red-brick council housing (on right-hand side) and new builds on the left. (a, b, and d from https://www.geograph.org.uk/ and licensed for reuse under the Creative Commons Attribution-ShareAlike 2.0 Generic license **(CC BY-SA 2.0)**, c and e both courtesy Y Iles-Caven, 2019 & 2022).

Bristol city centre is predominantly made up of a mixture of large buildings dating from the Georgian period, and post-war reconstruction to replace those lost to bombing, decay or modernisation ( Figure 3). The Georgian houses are largely of single skin construction and often made of sandstone, leading to an increased risk of damp and crumbling stone due to its porous nature. Buildings from the Victorian period radiating outwards from the centre in time to a predominance of housing built in the inter-war period and more modern housing in the suburbs (See Figure 4). The changing priorities of housing developers and in building standards over time result in each housing style bringing its own challenges ( Hashemi, 2013).

Bristol suffered heavy bombing during the Second World War, when almost 85,000 homes were destroyed. It was a target due to its proximity to Filton airfield and the Bristol Aeroplane Company based there (which built the Blenheim bomber and Beaufighter); the harbour in the city centre and the docks at Avonmouth ( Historic England, 2022). After the war there was a huge need for new homes to rehouse families and returning military personnel ( Drew, 2022).This resulted in massive development of the outer edges of Bristol where much of the housing dates from the 1940s to 1980s with the timeline predominantly radiating outwards from the centre. Destroyed buildings were also replaced leading to a snaggle toothed skyline ( Figure 3) where the Georgian/Victorian buildings are interspersed with post-war buildings in noticeably different styles.

Parts of Bristol still retain the old prefabricated temporary homes erected to house bombed-out families ( Drew, 2022) (see Figure 4). They provided homes for young families and also employment for factory workers who needed new items to manufacture. Many of these ‘prefabs’ remain despite the City Council’s attempts to replace them with more modern housing. This is partly due to historic importance and the fondness inhabitants have for their homes and the communities that have built up around them ( BBC News England, 2001). 

### Standard of housing

According to Ige *et al.* (2019) in 2015, 19% of the houses in the UK were not considered to be of a decent standard. They were not adequately maintained, did not have a reasonable standard of facilities and services nor were they appropriately thermally regulated. Older buildings are more at risk of poor standards ( Hetreed, 2008). The Joint Strategic Needs Assessment Health and Wellbeing Profile 2022/23 ( 2022) showed that in Bristol, children living in the older, more affluent areas of the city were least likely to experience poverty. Therefore, whilst ownership of Georgian/Victorian houses in affluent areas might be considered an indicator of wealth, these houses were built to last but fall short of modern standards with expensive upgrades required for damp, for crumbling masonry and installation of double-glazing.

Whilst these older homes may look impressive and are certainly valuable, and the city remains a desirable place to live ( Hawker, 2022), the ability to buy and run such large homes has meant that many have been split into flats/apartments or Houses of Multiple Occupation (HMOs). Due to complex preservation requirements (see Bristol City Council 2022a; Bristol City Council 2022b)and private landlords wishing to keep costs low, many of these buildings, in the historic heart particularly, remain under-developed with damp problems, inadequate heating and absence of double-glazing. Historic buildings are traditionally less energy efficient than new builds ( Moran *et al.,* 2012). The lack of heat retention and rising heating costs lead to families inadequately ventilating the home. For HMOs, Bristol City Council have recently introduced a licensing scheme ( Bristol City Council, 2022c), whereby landlords must register any HMOs to enable inspection to ensure living standards are acceptable.

### Housing outside Bristol

Many of the ALSPAC families live in the surrounding small towns (1991 population) such as the coastal towns south-west of Bristol: Weston-Super-Mare (77,000), Clevedon (21,275), Portishead (22,400), with Nailsea (15,500) to the south; and Yate/Chipping Sodbury (2011 pop. 35,000), Thornbury (2011 pop. 12,000) to the north, as well as in more rural areas. The housing in these towns is similarly mixed. The seaside resorts of Weston-Super-Mare, Clevedon and Portishead became popular in the Victorian period ( Roethe, 2020). These areas developed hugely post-war, along with old villages such as Thornbury and Chipping Sodbury with newer homes dating predominantly from the 1960s through to the 1980s. Yate, however, is considered a “new town”. Sparsely populated up until the post-war period, a huge amount of building has occurred since the 1960s.

## Methods

### ALSPAC sample

Pregnant mothers (n=14,541) resident in the former county of Avon in South-West England were recruited into the ALSPAC study. These mothers all had an expected delivery date between 1 ^st^ April 1991 to 31 ^st^ December 1992 and usually delivered at one of the three main maternity units (St. Michael’s (central Bristol), Southmead (north Bristol) and Weston-Super-Mare General Hospital). Of the initial 14,541 pregnancies enrolled, there were a total of 14,676 fetuses and 14,062 live births. Of these children, 13,988 were still alive at 1 year of age. Mothers were considered enrolled if they had returned at least one questionnaire or attended a “Children in Focus” clinic by 19 ^th^ July 1999. At the age of 7, the study team reached out to eligible mothers who had initially not joined the study and recruited additional families in order to boost numbers. Therefore, from age 7 the total sample number is 15,454 pregnancies, resulting in 15,589 fetuses of which 14,901 were alive at 1 year of age ( Boyd *et al.*, 2013; Fraser *et al.*, 2013; Northstone *et al.*, 2019).

### Data collection

Data has primarily been collected via multiple, self-completion questionnaires, which were sent to the study mother, at frequent intervals during pregnancy and throughout the study child’s life. Detailed data were collected on the types and size of housing, ownership status, frequency of household moves, living with parents and becoming homeless. Other reports of housing conditions in childhood were reported by parents using free text (this can be made available to researchers for coding on request). 

Paper-based questionnaires were administered between 1990 and 2011 via post. In accordance with the advice of the ALSPAC Ethics and Law Committee, partners were only included in the study if the mothers wished to include them. Questionnaires were sent to the mother who then passed the questionnaire on to the partner with a separate pre-paid return envelope. This method meant that ALSPAC was not able to follow-up or communicate directly with the partners ( Boyd *et al.,* 2013; Fraser *et al.,* 2013). Therefore, the numbers of partners’ questionnaires returned were less than those received for the mothers. Around 75% of mothers had partners who participated in the study. 

Each questionnaire and associated dataset were given a letter as an identifying prefix. The variable naming convention for these datasets uses the letter prefix followed by the unique number of the variable.

Table 1 shows the time points each of the parents’ questionnaires were administered that included questions on housing. Questionnaires A, B, C, D, PA and PB were completed during pregnancy (see Iles-Caven *et al.*, 2020) for full details). Post-delivery, questionnaires were sent to parents at regular intervals until the child was 18 years of age.

**Table 1.** Timeline of questionnaires concerning housing sent to the study child’s parents.

Mothers Questionnaires		Partners Questionnaires	
Questionnaire prefix	Approximate age of study child	Questionnaire prefix	Approximate age of study child
A *	8–28 weeks gestation	PA *	12 weeks gestation
B *	18–23 weeks gestation	PB *	18 weeks gestation
C *	32–40 weeks gestation	PC	8 weeks
D *	14–37 weeks gestation	PD	8 months
E	8 weeks	PE	21 months
F	8 months	PF	33 months
G	21 months	PG	47 months
H	33 months	PH	61 months
J	47 months	PJ	73 months
K	61 months	PK	85 months
L	73 months		
M	85 months	PM	110 months
P	110 months	PP	134 months
Q	122 months		
R	134 months		
T	18 years		

*Questionnaires administered depending on the week of gestation of enrolment

All of the ALSPAC questionnaires can be viewed here. The ALSPAC team have provided a useful Questionnaire Topic guide which summarises the information contained in each questionnaire.

A fully searchable data dictionary and variable search tool exist on the study website. This tool provides an easy identification tool for researchers looking for variables relating to a specific topic over the length of the study. 

## Results

### Types of housing

Table 2 describes the type of housing the mother lives in: detached, or semi-detached homes, terraced housing, apartments (flats), rooms and other types of accommodation (this has not been coded but is available in text format on request). The proportion living in detached homes increased steadily over time whereas those in self-contained apartments decreased.

**Table 2.** The type of home.

Variable	N	Whole Detached	Whole Semi- Detached	End of Terrace	Terrace	Self-Contained Flat	Rooms	Other ^a^
a008	13,285	1,915 (14.4%)	4,573 (34.4%)		4,284 (32.3%)	2,117 (15.9%)	196 (1.5%)	200 (1.5%)
f306	11,097	1,865 (16.8%)	4,080 (36.8%)		3,526 (31.8%)	1,412 (12.7%)	74 (0.7%)	140 (1.3%)
g354	10,191	1,981 (19.4%)	3,489 (34.2%)	992 (9.7%)	2,567 (25.2%)	1,062 (10.4%)	17 (0.2%)	83 (0.8%)
h264	9,539	2,064 (21.6%)	3,422 (35.9%)	926 (9.7%)	2,296 (24.1%)	761 (8.0%)	9 (0.1%)	61 (0.6%)
k5030	8,903	2,256 (25.3%)	3,330 (37.4%)	876 (9.8%)	1,934 (21.7%)	426 (4.8%)	8 (0.1%)	73 (0.8%)
m2030	8,267	2,426 (29.4%)	3,092 (37.4%)	773 (9.4%)	1,643 (19.9%)	262 (3.2%)	5 (0.1%)	66 (0.8%)
pk2030	4009	1360 (33.9)	1497 (37.3)	336 (8.4)	713 (17.8%)	73 (1.8%)	<5 (0.1%)	26 (0.7)
q2040	8,013	2,641 (33.0%)	3,047 (38.0%)	723 (9.0%)	1,370 (17.1%)	165 (2.1%)	<5 (0.0%)	65 (0.8%)

^a^ Details of the other types of accommodation (e.g. caravan, boat) are available as text.

In Table 3 and Table 4 the lowest floors of the accommodation and the number of rooms used for living and sleeping are summarised.

**Table 3.** The range in lowest levels of living accommodation (the median at all time points was the ground-level floor).

Variable	Time point	N	Range
a009	Pregnancy	13,251	Basement – 16th
f307	8m	10,785	Basement – 19th
g355	21m	10,029	Basement – 23rd
h265	33m	9,402	Basement – 29th
k5040	5y	8,753	Basement - 15th
m2040	7y	8,176	Basement - 9th
pk2040	7y	3,990	Basement – 9th
q2050	10y	7,952	Basement – 10th

**Table 4.** Numbers of rooms in the home used for living or sleeping.

Variable	Time point	No. rooms	N	Range
a043	Pregnancy	No. living rooms excluding kitchen	13,291	0–11
a044	Pregnancy	No. bedrooms	13,253	0–9
a045	Pregnancy	Total rooms	13,184	0–18
f343	8m	No. excluding kitchen	11,091	0–12
f344	8m	No. bedrooms	11,054	0–9
f345	8m	Total rooms	11,034	0–19
g413	21m	No. excluding kitchen	10,006	0–17
h293	33m	No. excluding kitchen	9,501	0–18
h294	33m	Total rooms	9,501	1–19
k5070	5y	No. excluding kitchen	8,874	0–18
m2070	7y	No. excluding kitchen	8,181	1–19
pk2070	7y	No. excluding kitchen	3,458	1 – 30
q2080	10y	No. excluding kitchen	7,616	1–23

Features of the home that were recorded include the type of kitchen (and whether it is large enough for the family to sit and eat), whether there is an indoor flushing toilet, a garden or a balcony. For each of these features, the mother reported whether or not the family has sole use or whether it was shared with others ( Table 5a– Table 5e). It should be noted that the details of whether there is a kitchen without room to sit and eat are not always consistent with the details of the presence of a kitchen with such facilities ( Table 5a and Table 5b); this is sometimes due to a failure to respond to the second question having answered the first but may also indicate that there is more than one kitchen in some homes.

**Table 5a.** Presence of a separate kitchen large enough for the family to sit and eat.

Variable	Time point	N	% sole use	% shared	% do not have
a040	pregnancy	13,346	47.0	2.7	50.3
f340	8m	11,213	49.5	1.1	50.5
g410	21m	9,957	52.9	0.8	46.4
h290	33m	9,571	54.0	0.5	45.5
k5060	5y	8,704	61.5	0.5	38.0
m2060	7y	8,044	63.7	0.4	35.8
pk2060	7y	3,895	64.8	0.5	34.7
q2070	10y	7,760	66.9	0.5	32.7

* Maternal reports in black, mother’s partner reports in red

**Table 5b.** Presence of a separate smaller kitchen in which there is insufficient space for the family to sit and eat.

Variable	Time point	N	% sole Use	% shared	% do not have
a041	pregnancy	13,328	54.4	2.8	40.8
f341	8m	11,121	61.8	1.2	37.1
g411	21m	8.404	72.3	0.8	26.9
h291	33m	9,571	56.9	0.5	42.7
k5061	5y	7,485	70.9	0.6	28.6
m2061	7y	6,575	68.0	0.6	31.4
pk2061	7y	3,299	65.8	0.5	33.7
q2071	10y	6,050	68.4	0.4	31.2

**Table 5c.** Presence of an indoor flushing toilet.

Variable	Time point	N	% sole use	% shared	% None
a042	pregnancy	12,765	92.7	5.2	2.1
f342	8m	10,869	95.3	2.1	2.6
g412	21m	10,062	97.5	1.2	1.4
h292	33m	9,355	97.5	1.0	1.6
k5062	5y	8,859	98.9	0.7	0.4
m2062	7y	8,133	98.9	0.5	0.5
pk2062	7y	3,935	98.5	0.7	0.8
q2072	10y	7,834	98.1	0.6	0.6

**Table 5d.** Presence of a garden or yard.

Variable	Time point	N	% sole use	% shared	% None
a049	pregnancy	13,256	82.6	9.9	7.5
f349	8m	11,139	87.6	6.2	6.3
g419	21m	10,192	90.8	4.5	4.8
h299	33m	9,560	92.9	3.7	3.1
k5069	5y	8,884	95.7	2.3	2.0
m2069	7y	8,269	97.2	1.9	0.9
pk2069	7y	4015	97.8	1.6	0.6
q2079	10y	8,005	97.9	0.1	2.0

**Table 5e.** Presence of a balcony.

Variable	Time point	N	% sole use	% shared	% none
a050	pregnancy	13,432	6.5	1.0	92.5
f350	8m	11,139	6.7	0.7	92.6
g420	21m	10,058	5.2	0.5	94.3
h300	33m	9,234	6.2	0.6	93.2
k5070	5y	8,560	6.1	0.4	93.4
m2070	7y	8,026	5.7	0.3	94.0
pk2070	7y	3,835	7.8	0.2	92.0
q2070	10y	7,726	5.9	0.1	93.9

### Financial aspects

Housing tenure distinguishes between homes that were being bought (mortgaged), were owned outright, rented from the Council (public housing), rented from a non-profit housing association, or rented from a private landlord ( Table 6).

**Table 6.** Housing tenure: Numbers (%) in each category of housing.

Variable	N	Mort	BBC	OO	RC	PRF	PRUF	HAR	Other
a006	13,335	9,469 * (71.0)	288 (2.2)	-	1,918 (14.4)	575 (4.3)	395 (3.0)	220 (1.7)	470 (3.5)
f304	11,091	8,299 * (74.8)	246 (2.2)	-	1,485 (13.4)	249 (2.3)	354 (3.2)	180 (1.6)	278 (2.5)
g352	10,204	7,691 (75.4)	209 (2.1)	96 (0.9)	1,278 (12.5)	195 (1.9)	340 (3.3)	180 (1.8)	215 (2.1)
h262	9,579	7,290 (76.1)	220 (2.3)	117 (1.2)	1,115 (11.6)	148 (1.6)	297 (3.1)	196 (2.1)	196 (2.1)
k5010	8,898	6,877 (77.3)	74 (0.8)	254 (2.9)	960 (10.8)	105 (1.2)	265 (2.4)	217 (2.4)	146 (1.6)
m2010	8,275	6,535 (79.0)	81 (1.0)	334 (4.0)	714 (8.6)	61 (0.7)	230 (2.8)	180 (2.2)	140 (1.7)
pk2040	4018	3426 (85.3)	33 (0.8)	168 (4.2)	170 (4.2)	28 (0.7)	84 (2.1)	37 (0.9)	72 (1.8)
q2010	8,031	6,280 (78.2)	127 (1.6)	527 (6.6)	548 (6.8)	33 (0.4)	209 (2.6)	197 (2.5)	110 (1.4)
t1010	4,154	2,770 (66.7)	12 (0.3)	953 (22.9)	120 (2.9)	20 (0.5)	134 (3.2)	89 (2.1)	56 (1.4)

* Mortgaged included owned outright Mort= mortgaged; BBC = being bought from the Council; OO = Owned outright; RC = Rented from the Council; PRF = private rented, furnished; PRUF = private rented unfurnished; G = Housing Association rented; Other = eg. Boat, caravan (details available as text)

All homes are expected to pay a Council Tax, the amount (known as the tax band) being related to the size, layout, character, location and value of the home. Bands are based on the rateable value of the property as of 1 ^st^ April 1991 and rise on average about 2–3% per annum. The current (2022) tax amounts per annum for Bristol City Council range from the lowest band, A (£1,487) to the highest H/I (£4,461). Other councils covering the ALSPAC area are South Gloucestershire and North Somerset, which have slightly lower rates in 2022 than Bristol. This tax covers the costs of the police, education, social care and facilities such as waste and recycling collection, local libraries and upkeep of public spaces, etc. The Council Tax bands of the homes in which the mothers were living when the study children were aged 10 and 18 are shown in Table 7.

**Table 7.** The Council Tax band of the study participants’ homes (A is the lowest; H/I the highest).

Variable	N	A	B *	C *	D	E	F	G	H/I
q2020	5,178	233 (4.5%)	1,319 (25.5%)	1,241 (24.0%)	1,169 (22.6%)	607 (11.7%)	335 (6.5%)	230 (4.4%)	44 (0.9%)
t1013	2,750	83 (3.0%)	550 (20.0%)	652 (23.7%)	713 (25.9%)	358 (13.0%)	216 (7.9%)	146 (5.3%)	32 (1.2%)

*The median Council Tax band in Bristol is B, and C in S Glos and N Somerset.

Information was collected on whether the parents reported having difficulty in paying for their accommodation. Table 8 shows that the proportion stating they had difficulties reduced over time.

**Table 8.** How difficult it is to pay the rent or mortgage.

Variable Name	N	Time point	Very difficult	Fairly difficult	Some difficulty	Not difficult	Paid by Social Security #
c523	11,999	Pregnant	4.6%	7.7%	17.9%	69.7%	NA
f803	11,114	8 m	4.8%	8.4%	19.3%	67.6%	NA
pd683	7,052		5.5%	9.3%	22.9%	62.3%	NA
g838	10,065	21m	3.3%	7.2%	16.6%	64.7%	8.2%
pe463	6,066	21m	3.4%	6.9%	19.1%	66.4%	4.3%
k6203	8,772	5 years	1.6%	4.2%	13.8%	72.0%	8.5%
r9003	7,426	11 years	1.1%	2.6%	10.3%	76.1% *	NA
pp9003	3,558	11 years	0.7%	2.1%	10.6%	81.1%	5.5%

*a further 10.1% stated that they were not responsible for paying; NA = not asked # Monetary assistance from the state for people with an inadequate or no income.

### Stability of the home

Many of the mothers moved home before and during the child’s life: three-quarters had moved in the 5 years prior to the pregnancy, and subsequently over 10% had moved during each of the first 11 years of the child’s life ( Table 9). In parallel there were small numbers of mothers and their partners who had been homeless during the pregnancy and subsequently, this reduced over time ( Table 10).

**Table 9.** Moving home.

Moved during period	Variable name	Proportion (N) moved	Other relevant variables
From 5 years before pregnancy	a005	75.9% (9917)	a003-5 ^a b^
	pa005	74.6% (6239)	pa003-5 ^a b^
1 ^st^ half of pregnancy	b591	10.2% (1214)	
	pb181a	10.7% (1040)	
1 ^st^ 7m of pregnancy	c470	13.8% (1654)	c471 ^a^
Mid-pregnancy to 4wk PP	e421	12.2 % (1418)	
Mid-pregnancy to 8 wks PP	pc221	11.7% (972)	
In 1 ^st^ 8 months PP	f302	14.1% (1523)	f300-3a ^a b^
	pd241	10.8% (758)	
8 –21m	g321a	16.7% (1718)	g350-1 ^a b^
	pe321a	15.1% (927)	
18 – 33m	h231	19.9% (1903)	h260-1 ^a b^
	pf5021	18.5% (998)	
30 – 47m	j321	19.6% (1878)	
	pg3021	17.7% (900)	
4 – 5y	k4021a	13.1% (1170)	k5000-1 ^a b^
	ph4021	12.8% (585)	
5 – 6y	l4021	12.7% (1079)	
	pj4021	12.7% (562)	
At 7y			m2000-1 ^a b^
			pk2000-1 ^a b^
6 – 9y	p4021	24.2% (1896)	
	pm2021	23.0% (831)	
At 10y			q2001 ^a^
9 – 11y	r5021	16.9% (1276)	
	pp5021	16.0% (578)	
17 – 18y	t3320	4.4% (181)	t1000-5 ^a b^

^a^No. of moves in period of time ^b^Age or date at last move PP = postpartum

**Table 10.** Made homeless.

Became homeless during period	Variable name	Proportion (n) homeless
1 ^st^ half of pregnancy	b593	1.7% (205)
	pb183	1.2% (121)
1 ^st^ 7m of pregnancy	c472	2.3% (268)
Mid-pregnancy to 4wk PP	e423	1.3% (148)
Mid pregnancy to 8weeks PP	pc223	1.1% (88)
First 8 months PP	f243a	2.2% (125)
	pd243	0.7% (52)
8 –21m	g323a	1.0% (105)
	pe323	0.5% (30)
30 – 47 m	j323	0.8% (78)
	pf5023	0.7% (39)
4 – 5y	k4023a	0.5% (47)
	pg3023	0.5% (25)
5 – 6y	l4023	0.6% (49)
	ph4023	0.3% (15)
6 – 9y	p4023	0.8% (62)
	pj4023	0.4% (16)
9 – 11y	r5023	0.5% (35)
	pm2023	0.3% (12)
17 – 18y	t3322	0.1% (5)

PP = postpartum

Not all families were living in their own home – during pregnancy 6% were living in their parents’ home, but this reduced to around 1% by the time the children were aged over 2 years, and less still as time went on ( Table 11).

**Table 11.** Whether the mother lives in her own home, with her parents or with others.

Variable name	Time point	N	Own Home	Partner’s Home	Parent’s Home	Partner’s Parents Home	Other
a007	Pregnancy	13,232	11,747 (88.8%)		801 (6.1%)		684 (5.2%)
f305	8m	11,049	10,432 (94.4%)		355 (3.2%)		262 (2.4%)
g353	21m	10,146	9,698 (95.6%)	119 (1.2%)	151 (1.5%)	32 (0.3%)	146 (1.4%)
h263	33m	9,489	9,116 (96.1%)	100 (1.1%)	103 (1.1%)	20 (0.2%)	147 (1.6%)
k5020	5y	8,572	8,572 (97.2%)	66 (0.8%)	64 (0.7%)	18 (0.2%)	102 (1.2%)
m2020	7y	8,243	7,991 (96.9%)	99 (1.2%)	51 (0.6%)	14 (0.2%)	88 (1.1%)
pk2020	7y	4002	3856 (96.4%)	76 (1.9%)	12 (0.3%)	7 (0.2%)	51 (1.3%)
q2030	10y	7,979	7,793 (97.7%)	58 (0.7%)	33 (0.4%)	6 (0.1%)	89 (1.1%)

### Strengths and limitations

The strengths of this data are the ALSPAC dataset is large with over 14,000 enrolled in the study ( Boyd *et al.,* 2013). The only inclusion requirements at enrolment for this study were the geographical location of residence and the expected date of delivery. The participants recruited to the study were broadly representative of the general population of new parents resident in the area at the time in terms of sex, ethnicity and socio-economic status ( Fraser *et al*., 2013).

All parent participants included at birth received the same questions and one of the major strengths of this study is that the housing questions are linkable to other data collected throughout the family’s inclusion in the study. This includes information about their relationships with the study child, biological markers from the study child, data regarding their parent’s health, life experiences and demographics and data gathered from their grandchildren. This makes the data very flexible and relatable to intergenerational aspects of the family’s life.

A limitation of this study is the lack of diversity, because at the time of enrolment, the county of Avon was mainly Caucasian, therefore there were too few Black, Asian and Minority Ethnic (BAME) participants (<6% in all) to allow for detailed analysis by ethnic background.

## Data availability

ALSPAC data access is through a system of managed open access. The steps below highlight how to apply for access to the data included in this paper and all other ALSPAC data.

1. Please read the ALSPAC access policy which describes the process of accessing the data and biological samples in detail, and outlines the costs associated with doing so.
2. You may also find it useful to browse our fully searchable research proposals database, which lists all research projects that have been approved since April 2011.
3. Please submit your research proposal ( https://proposals.epi.bristol.ac.uk/) for consideration by the ALSPAC Executive Committee using the online process. You will receive a response within 10 working days to advise you whether your proposal has been approved.
If you have any questions about accessing data, please email: alspac-data@bristol.ac.uk (data) or bbl-info@bristol.ac.uk (samples).

### Ethical approval and consent

Prior to commencement of the study, approval was sought from the ALSPAC Ethics and Law Committee and the Local Research Ethics Committees. Consent for the use of data collected via questionnaires and clinics was implied from participants following the recommendations of the ALSPAC Ethics and Law Committee at the time. Questionnaires were completed in the participants own home and the return of the questionnaires was taken as continued consent for their data to be included in the study ( Birmingham, 2018). Full details of the approvals obtained are available from the study website. Study members have the right to withdraw their consent for elements of the study or from the study entirely at any time.
