# The external housing environment of participants in the Avon Longitudinal Study of Parents and Children (ALSPAC): a resource for studies of influences on health

**DOI:** 10.12688/wellcomeopenres.17996.2

**Published:** 2022-12-21

**Authors:** Genette Ellis, Yasmin Iles-Caven, Kate Northstone, Jean Golding

**Affiliations:** 1 Centre for Academic Child Health, Population Health Sciences, Bristol Medical School, University of Bristol, Oakfield House, Oakfield Grove, Bristol, BS8 2BN, UK; 2 Population Health Sciences, Bristol Medical School, University of Bristol, Oakfield House, Oakfield Grove, Bristol, BS8 2BN, UK

**Keywords:** ALSPAC, housing, environment, well-being, physical health, childhood, longitudinal cohort

## Abstract

**Background:** Early life environmental health exposures related to housing can have a significant impact on an individual’s physical and mental health and physical development. Housing exposures can fall into two main areas – a representation of social circumstances and physical conditions.

**Methods:** During pregnancy and post-delivery, self-completion questionnaires concerning the housing environment were administered to the ALSPAC (Avon Longitudinal Study of Parents & Children) study mothers and their partners until the study offspring were aged 18. Data collected included types of housing, housing tenure, numbers of rooms in the home, whether shared or sole use of kitchen and indoor flushing toilet, household moves, Council Tax band, difficulties in affording rent/mortgage and becoming homeless. Here we also describe the historic development of housing in the Bristol and surrounding Avon areas.

**Results:** Data collected included the tenure (e.g., owned/rented) of the home, its size (indexed by the number of rooms), the presence of over-crowding (measured by the number of residents per room), presence of amenities, and frequency of household moves. This information was collected on over 13,000 women during pregnancy >8000 at age 10 and >4000 at 18 years. Council Tax bands were asked at 10 and 18 years.

**Conclusions:** This is the first of two Data Notes on the housing type and housing circumstances of the families enrolled in ALSPAC. The second Data Note will detail their internal housing conditions. The data provides an excellent resource for researchers when considering the influences of housing on physical and mental health and development.

## Introduction

Housing quality and housing instability can have significant impacts on physical and psychological health, including increased instances of depression and anxiety in women ( Suglia *et al.*, 2011); morbidity in infancy (e.g. Baker *et al.*, 1998); asthma and allergies in childhood (e.g. Choi *et al.*, 2017); and general ill-health (e.g. Marsh *et al.*, 2000). Therefore, housing type may be an important consideration when looking at the prevalence of morbidity in parents and their offspring. In this paper we describe the data collected from families enrolled in the Avon Longitudinal Study of Parents and Children (ALSPAC), centred on the city of Bristol and surrounding areas in South-West England, regarding their housing type and circumstances during the first 18 years of life. To aid international readers, we provide a brief history of the area and the most common housing stock. The study provides an excellent resource for researchers when considering health and/or developmental outcomes of the study children and/or their parents over time. This includes data on the homes of the parents in childhood (e.g. H questionnaire: Your Health, Events & Feelings). It would assist researchers to also refer to the paper by Boyd and colleagues (2019) which describes data available in ALSPAC such as address histories, Index of Multiple Deprivation (IMD), geocoding, external air pollution, background radiation etc, and describes linkage to third-party records, whilst protecting confidentiality.

### Historical background and the manmade environment

There is evidence that Bristol and the surrounding areas of the former county of Avon have been inhabited by humans since the Stone Age, with various archaeological sites such as Stanton Drew stone circle ( David *et al.*, 2004) and barrows (e.g. the bowl barrow at Southmead), iron age hill forts (e.g. in Henbury), Clifton Hill and Kings Weston Hill camps and the Druid Stoke burial chamber (in Stoke Bishop) ( Ancient & Scheduled Monuments, 2022). The ancient settlement of *Brycgstow* (Old English: Place of the Bridge) grew up around the confluence of two rivers, the Frome and the Avon (which gave sea access via the River Severn at Avonmouth) before 1000 A.D. By 1066, the population was around 4000, centred around the. castle (built <1088, destroyed 1656) and within the walled town. The port was already important between the 11 ^th^ and 13 ^th ^centuries (in the 1240s the city port was expanded by diverting the river Frome into a deep canal (St. Augustine’s Reach). At this time, wine was the main import (from Spain, Portugal and Southwest France). Bristol exports included dyed woollen products, lead, sail cloth and rope. However, one of the exports to Dublin between the 10 ^th^ and 15 ^th^ centuries was Welsh and Northern English slaves. By the 1460s it was the country’s second port trading with Ireland, Iceland and Gascony. Around 1480, Bristol’s Society of Merchant Venturers began sponsoring exploration of the north Atlantic. Bristol attained city status in 1542 ( Wikipedia, 2022).

As such, the city centre’s secular buildings and those in the surrounding area range in date and style from the Tudor/Elizabethan (1485-1603), Georgian (1714-1836), Victorian (1837-1901) and Edwardian (1901-1910) eras, to inter-war housing through to the contemporary, with ongoing improvements, gentrification, repurposing, and new builds.

Much of Bristol’s housing grew up around the industries that flourished in the old city including soap, potteries and glass production, tobacco, sugar and chocolate, leather, textiles, lead shot manufacturing and breweries. These influenced the layout and expansion of housing across the city ( Harvey & Press, 1988), with an emphasis on cheap accommodation for employees, typically ‘two up-two down’ houses.

By far, the greatest impact on house building in the Georgian period was the trans-Atlantic slave trade. Between 1700 and 1804, 2000 Bristol ships carried an estimated 500,000 enslaved Africans to the New World. Indeed, in 1747, it was Britain’s busiest slaving port. The city also processed many of the slave-produced goods such as sugar and tobacco. The trade brought (directly or indirectly) great wealth to many merchants in the city, who moved out of the central port area and up the hill to rural Clifton where many of the elegant townhouses, crescents and villas they built can be found today. After Bristol’s decline as a slaving port, Clifton had one of the highest numbers of retired plantation owners in Britain ( Lewin-Turner, 2022). Many merchants with connections to the slave trade (e.g., Edward Colston, the Farr family, the Wills family) were also great philanthropists creating schools, alms houses and the Theatre Royal.

**Figure 1.**
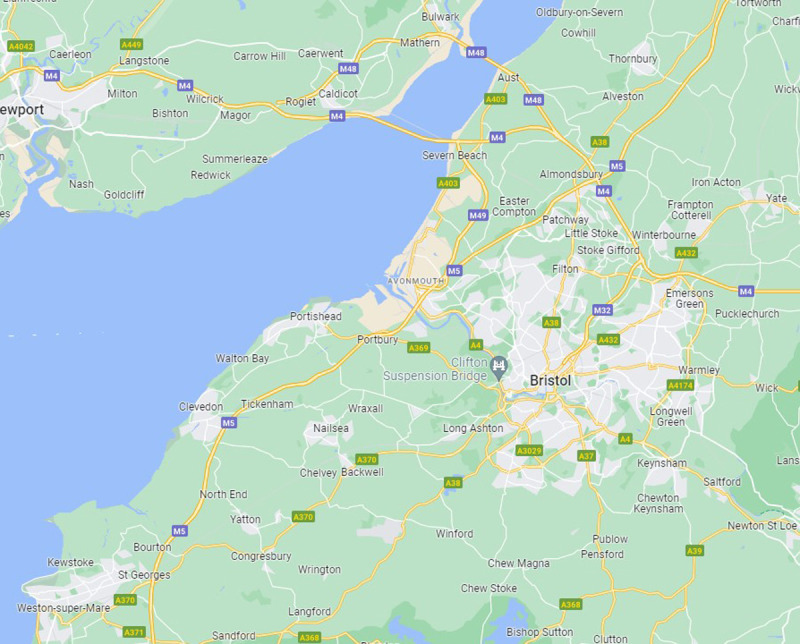
Avon area showing the main small towns and villages. Map credit: Google Maps.

Until 1949, there was extensive coal mining particularly to the north-east of Bristol (e.g., the Kingswood and Fishponds areas) and Bedminster to the south (see Figure 1), which led to the expansion of established villages where much of the original (Victorian) housing stock remains. Currently, the city has significant aerospace, media, information technology and financial services sectors, as well as tourism and education, with two universities with a combined student population of around 60,000.

**Figure 2.**
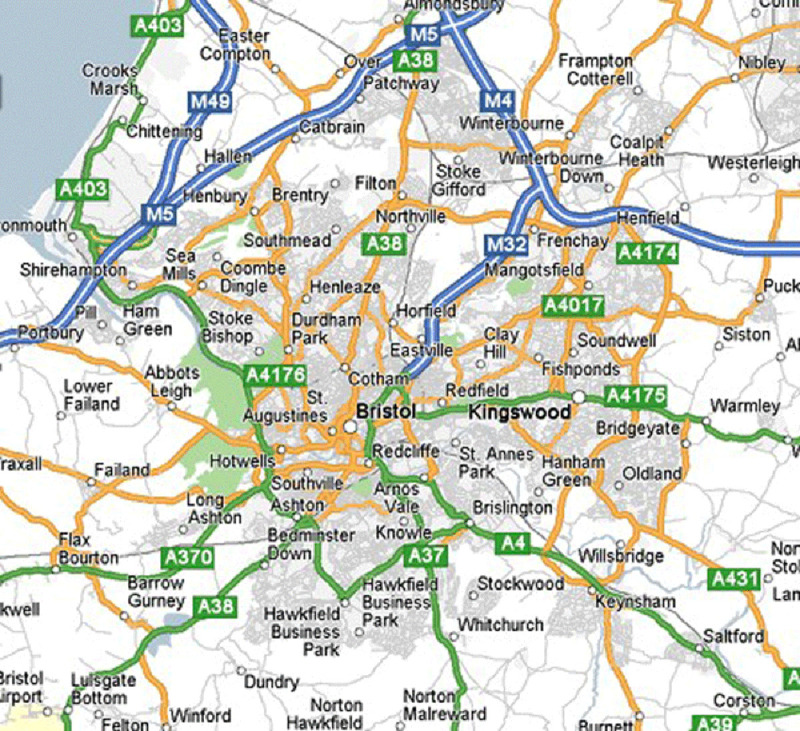
Map of Bristol and the surrounding areas indicating main arterial roads, suburbs and small villages within the ALSPAC catchment area. Map credit: https://gadgets2018blog.blogspot.com/2017/10/map-of-bristol-area.html.

The city stands at the junction of the M4 and M5 motorways, with the M32 allowing direct access to the city centre ( Figure 2). Like many old British cities, the road system was not designed to cope with current traffic volumes and peak commuter time congestion. This has raised noise pollution ( BBC News England, 2015) and air quality ( Clean Air for Bristol, 2022) concerns. As of 28 ^th^ November, 2022 Bristol City Council has introduced a ‘Clean Air Zone’ (see Bristol’s Clean Air Zone).

### The housing stock

**Figure 3.**
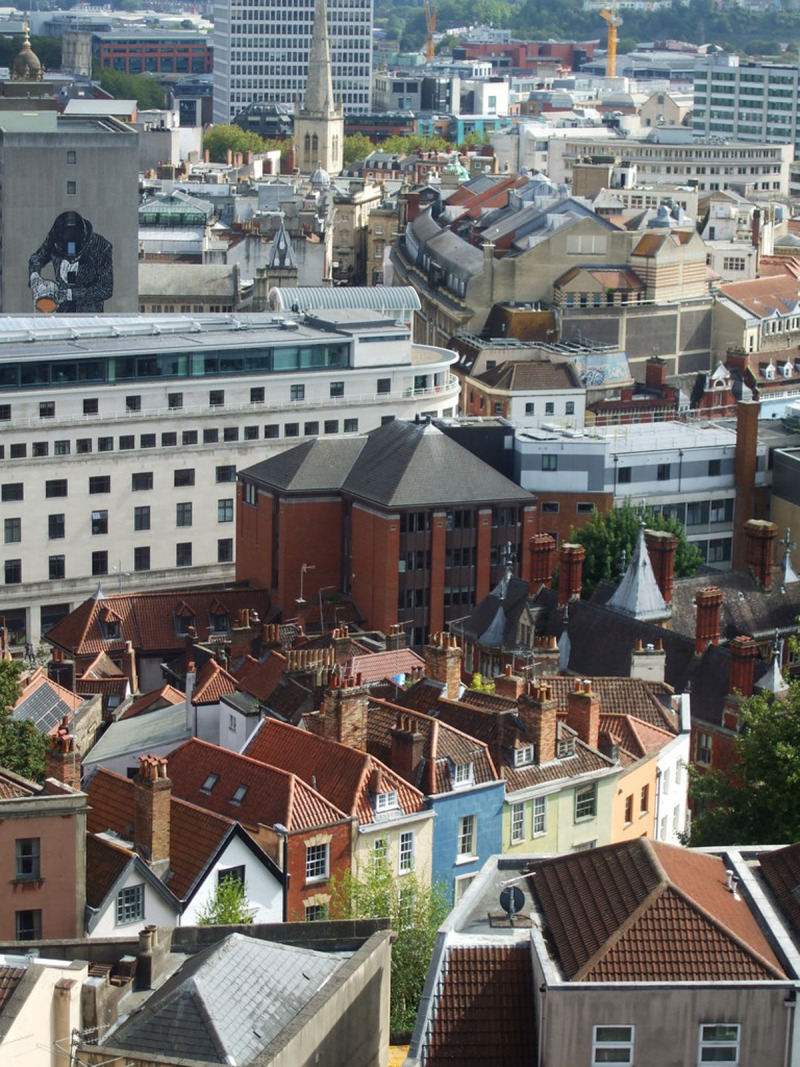
ST5873 : From the tower of St Michael on the Mount Without: Bristol city centre skyline 2019 showing modern tower blocks, ancient churches interspersed with Georgian and Victorian houses (now largely given over to offices). © Copyright Neil Owen and licensed for reuse under the Creative Commons Attribution-ShareAlike 2.0 Generic license (CC BY-SA 2.0).

**Figure 4.**
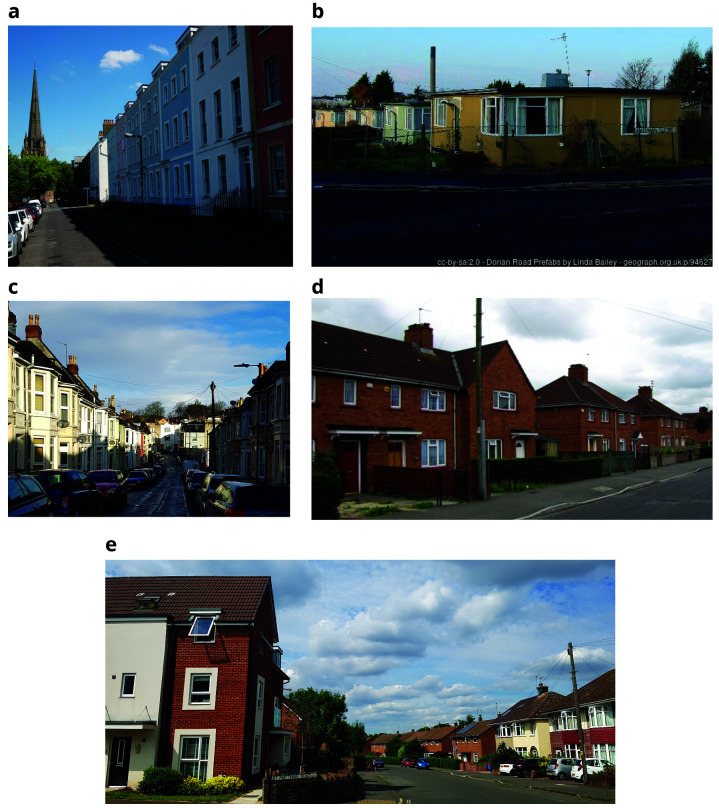
Typical Bristol housing (Top row, left: **a**. elegant Georgian terrace in central Bristol © David Hallam-Jones right: **b**. Prefab’ homes © Copyright **Linda Bailey**. Bottom row, left: **c**. Victorian terraced street; right: **d**. 1930s Council (social) housing © Copyright Jez McNeill; **e**. Bristol suburban street showing 1930s semi-detached homes, 1950s red-brick council housing (on right-hand side) and new builds on the left. (a, b, and d from https://www.geograph.org.uk/ and licensed for reuse under the Creative Commons Attribution-ShareAlike 2.0 Generic license **(CC BY-SA 2.0)**, c and e both courtesy Y Iles-Caven, 2019 & 2022).

Bristol city centre is predominantly made up of a mixture of large buildings dating from the Georgian period, and post-war reconstruction to replace those lost to bombing, decay or modernisation ( Figure 3). The Georgian houses are largely of single skin construction and often made of sandstone, leading to an increased risk of damp and crumbling stone due to its porous nature. Buildings from the Victorian period radiating outwards from the centre in time to a predominance of housing built in the inter-war period and more modern housing in the suburbs. These are illustrated in Figure 4 and show the diversity of ALSPAC homes. ALSPAC respondents have also reported living in caravans, houseboats and ‘prefabs’. The changing priorities of politicians, housing developers and in building standards over time result in each housing style bringing its own challenges ( Hashemi, 2013).

Following the Housing & Town Planning Act of 1919, local councils became the main providers of lower cost housing and were providing ‘garden estates’ in the suburbs of 3-bedroom family homes of around 1000sq feet. However, these were largely rented to more affluent working families. By 1924, a need for cheaper, smaller housing (~620sq feet) was required to house those displaced by increasing slum clearance. In Bristol, for example, there was a need to rehouse 25,000 inhabitants residing in 5000 unfit dwellings. The 1930 Housing Act stimulated further slum clearance with heavy subsidies to councils building social housing. Council houses were most usually built of red bricks in a certain style and there are usually at least four of these houses in every village.

Bristol suffered heavy bombing during the Second World War, when almost 85,000 homes were destroyed. It was a target due to its proximity to Filton airfield and the Bristol Aeroplane Company (which built the Blenheim bomber and Beaufighter); the harbour in the city centre, the docks at Avonmouth and Portishead, and was an important railway junction. The outer suburbs and rural areas also suffered from bombing e.g. Parnall Aircraft works at Yate, Portishead docks and Weston-super-Mare. Although no area was left unscathed, and the city centre suffered severely, the residential suburbs most affected included Fishponds, Eastville, Whitehall, Easton, Redcliffe, Bedminster, Clifton, Redland, Cotham and Redland ( Bristol during World War 2; Historic England, 2022). Post-war there was a huge need for new homes to rehouse families and returning military personnel ( Drew, 2022). This resulted in massive development of the outer edges of Bristol where much of the housing dates from the 1940s to 1980s with the timeline predominantly radiating outwards from the centre. The same pattern can be seen in small towns such as Weston-super-Mare, Clevedon, Nailsea, Yate and Thornbury. Destroyed buildings in the city centre were also replaced leading to a snaggle toothed skyline ( Figure 3) where the Georgian/Victorian buildings are interspersed with post-war buildings in noticeably different styles.

Parts of Bristol still retain the old prefabricated (‘prefabs’) temporary homes erected to house bombed-out families ( Drew, 2022) (see Figure 4), and in fact has the highest number of prefabs (330) still remaining in England. They provided homes for young families and also employment for factory workers who needed new items to manufacture. Many of these ‘prefabs’ remain despite the City Council’s attempts to replace them with more modern housing. This is partly due to historic importance and the fondness inhabitants have for their homes and the communities that have built up around them ( BBC News England, 2001). 

Post-war expansion of council housing during the 1950s and 1960s consisted of the development of more peripheral estates on or close to the edge of the city. By the 1970s the council was building fewer homes and concentrated on upgrading and maintaining their current stock. The Housing Act of 1980 enabled eligible council tenants the ‘right-to-buy’ their homes at up to 60% off the market value and many did so. Since the 1990s there has been increasing reliance on private sector building developments to include a percentage of ‘affordable’ housing, managed by Housing Associations. For a more detailed overview see The History of Council Housing (uwe.ac.uk) (2008).

### Standard of housing in Bristol

According to Ige *et al.* (2019) in 2015, 19% of the houses in the UK were not considered to be of a decent standard. They were not adequately maintained, did not have a reasonable standard of facilities and services nor were they appropriately thermally regulated. Older buildings are more at risk of poor standards ( Hetreed, 2008). The Joint Strategic Needs Assessment Health and Wellbeing Profile 2022/23 (2022) showed that in Bristol, children living in the older, more affluent areas of the city (e.g. Clifton, Redland, Cotham and Kingsdown) were least likely to experience poverty, whilst those living in similarly aged dwellings in more deprived areas such as St. Pauls and St Werburghs had poorer quality housing. Therefore, whilst ownership of Georgian/Victorian houses in affluent areas might be considered an indicator of wealth, these houses were built to last but fall short of modern standards, with expensive upgrades required for treatment of damp, for crumbling masonry, lead pipe replacement and installation of double-glazing. The majority of privately owned Georgian/Victorian homes in the affluent areas have been upgraded; and some (often in the private rental sector) had been poorly maintained in the past, but are now much better regulated, with fire doors, safety certification and improved insulation. Whilst these older homes may look impressive, are certainly very valuable, and the city remains a desirable place to live ( Hawker, 2022), the ability to buy and run such large homes has meant that many have been split into flats/apartments or Houses in Multiple Occupation (HMOs – these are defined as a building/flat where more than one household share basic amenities such as a toilet, bathroom or kitchen). Due to complex preservation requirements (see Bristol City Council 2022a; Bristol City Council 2022b) and private landlords wishing to keep costs low, many of these buildings, in the historic heart particularly, remain under-developed with damp problems, inadequate heating and absence of double-glazing. Historic buildings are traditionally less energy efficient than new builds ( Moran *et al.,* 2012). The lack of heat retention and rising heating costs lead to families inadequately ventilating the home. For HMOs, Bristol City Council have recently introduced a licensing scheme ( Bristol City Council, 2022c), whereby landlords must register any HMOs to enable inspection to ensure living standards are acceptable.

### Housing outside Bristol

About 25% of the ALSPAC families live in the surrounding small towns (with 1991 populations) such as the coastal towns south-west of Bristol: Weston-Super-Mare (77,000), Clevedon (21,275), Portishead (22,400); Nailsea (15,500) to the south; and Yate/Chipping Sodbury (2011 pop. 35,000), Thornbury (2011 pop. 12,000) to the north, as well as in more rural areas. The housing in these towns is of a similar mix and spread to most of Bristol. The seaside resorts of Weston-Super-Mare, Clevedon and Portishead became popular in the Victorian period ( Roethe, 2020). These areas developed hugely post-war, along with old villages such as Thornbury and Chipping Sodbury with newer homes dating predominantly from the 1960s through to the 1980s. Yate, however, is considered a “new town” sparsely populated up until the post-war period, a huge amount of building has occurred since the 1960s.

## Methods

### ALSPAC sample

Pregnant mothers (n=14,541) resident in the former county of Avon in South-West England were recruited into the ALSPAC study. These mothers all had an expected delivery date between 1 ^st^ April 1991 to 31 ^st^ December 1992 and usually delivered at one of the three main maternity units (St. Michael’s (central Bristol), Southmead (north Bristol) and Weston-Super-Mare General Hospital). Of the initial 14,541 pregnancies enrolled, there were a total of 14,676 fetuses and 14,062 live births. Of these children, 13,988 were still alive at 1 year of age. Mothers were considered enrolled if they had returned at least one questionnaire or attended a “Children in Focus” clinic by 19 ^th^ July 1999. At the age of 7, the study team reached out to eligible mothers who had not joined the study initially and recruited additional families in order to boost numbers. Therefore, from age 7 the total sample number is 15,454 pregnancies, resulting in 15,589 fetuses of which 14,901 were alive at 1 year of age ( Boyd *et al.*, 2013; Fraser *et al.*, 2013; Northstone *et al.*, 2019).

### Data collection

Data has primarily been collected via multiple, self-completion questionnaires, which were sent to the study mother, at frequent intervals during pregnancy and throughout the study child’s life. Detailed data were collected on the types and size of housing, ownership status, frequency of household moves, living with parents and becoming homeless. Other reports of housing conditions in childhood were reported by parents using free text (this can be made available to researchers for coding on request). These data were collected even if the respondent had moved out of area or abroad. Subsequently, record linkage to the NHS and National Statistics Postcode Lookup facility, linkage with third-parties’ physical environment records or social or built environment records enables geocoding. This includes the location of schools attended by the offspring. Some ALSPAC publications have relied on geocoded data or geospatial techniques; or used location-based data as covariates such as adjusting for social position using the Index of Multiple Deprivation (IMD); or used geographical areas to support multilevel modelling (see Boyd *et al.*, 2019 for more detail). For those wishing to research the broader external environment (e.g. proximity to main roads, green spaces and power lines) it is worth reading this paper in conjunction with Boyd *et al.*, 2019.

Paper-based questionnaires were administered between 1990 and 2011 via post. In accordance with the advice of the ALSPAC Ethics and Law Committee, partners were only included in the study if the mothers wished to include them. Questionnaires were sent to the mother who then passed the questionnaire on to the partner with a separate pre-paid return envelope. This method meant that ALSPAC was not able to follow-up or communicate directly with the partners ( Boyd *et al.,* 2013; Fraser *et al.,* 2013). Therefore, the numbers of partners’ questionnaires returned were less than those received for the mothers. Around 75% of mothers had partners who participated in the study. 

Each questionnaire and associated dataset were given a letter as an identifying prefix. The variable naming convention for these datasets uses the letter prefix followed by the unique number of the variable. For example, variable a008 is from questionnaire A, variable number 008. Variables from partner questionnaires use a double-letter prefix plus variable number (e.g. pk2030).

Table 1 shows the time points each of the parents’ questionnaires were administered that included questions on housing. Questionnaires A, B, C, D, PA and PB were completed during pregnancy (see Iles-Caven *et al.*, 2020) for full details). Post-delivery, questionnaires were sent to parents at regular intervals until the child was 18 years of age.

**Table 1.** Timeline of questionnaires concerning housing sent to the study child’s parents.

Mothers Questionnaires		Partners Questionnaires	
Questionnaire prefix	Approximate age of study child	Questionnaire prefix	Approximate age of study child
A *	8–28 weeks gestation	PA *	12 weeks gestation
B *	18–23 weeks gestation	PB *	18 weeks gestation
C *	32–40 weeks gestation	PC	8 weeks
D *	14–37 weeks gestation	PD	8 months
E	8 weeks	PE	21 months
F	8 months	PF	33 months
G	21 months	PG	47 months
H	33 months	PH	61 months
J	47 months	PJ	73 months
K	61 months	PK	85 months
L	73 months		
M	85 months	PM	110 months
P	110 months	PP	134 months
Q	122 months		
R	134 months		
T	18 years		

*Questionnaires administered depending on the week of gestation of enrolment

All of the ALSPAC questionnaires can be viewed here. The ALSPAC team have provided a useful Questionnaire Topic guide which summarises the information contained in each questionnaire.

A fully searchable data dictionary and variable search tool exist on the study website. This tool provides an easy identification tool for researchers looking for variables relating to a specific topic over the length of the study. 

## Results

One of the reasons Avon had been chosen as the site of the cohort study had been the relatively low levels of outward migration. During pregnancy both the mother and her partner were asked “How long have you lived in or near Avon?”, with the following possible responses: <1 year; 1–4 years; 5–9 years; 10+ years and whole life (variables a002 and pa002). For mothers, 53.4% had lived their entire life in the area compared with 50.9% of partners. Numbers for 10+ years were 16.9% and 20.4%; 5–9 years 11.1% and 11.9%; 1–4 years 13.6% and 13.5%; and for less than 1 year 5% and 3.3% respectively.

### Types of housing

Table 2 describes the type of housing the mother lives in: detached, or semi-detached homes, terraced housing, apartments (flats), room(s) and other types of accommodation (these have not been coded but are available in text format on request). The proportion living in detached homes increased steadily over time whereas those in terraced, self-contained apartments, room(s) and ‘other’ accommodation decreased.

**Table 2.** The type of home. (The variables comprise the questionnaire source and variable number (e.g. a008 is in the A questionnaire administered at 8–28 weeks gestation – see Table 1 for time of administration).

Variable	N	Whole Detached	Whole Semi- Detached	End of Terrace	Terrace	Self-Contained Flat	Rooms	Other ^a^
a008	13,285	1,915 (14.4%)	4,573 (34.4%)		4,284 (32.3%)	2,117 (15.9%)	196 (1.5%)	200 (1.5%)
f306	11,097	1,865 (16.8%)	4,080 (36.8%)		3,526 (31.8%)	1,412 (12.7%)	74 (0.7%)	140 (1.3%)
g354	10,191	1,981 (19.4%)	3,489 (34.2%)	992 (9.7%)	2,567 (25.2%)	1,062 (10.4%)	17 (0.2%)	83 (0.8%)
h264	9,539	2,064 (21.6%)	3,422 (35.9%)	926 (9.7%)	2,296 (24.1%)	761 (8.0%)	9 (0.1%)	61 (0.6%)
k5030	8,903	2,256 (25.3%)	3,330 (37.4%)	876 (9.8%)	1,934 (21.7%)	426 (4.8%)	8 (0.1%)	73 (0.8%)
m2030	8,267	2,426 (29.3%)	3,092 (37.4%)	773 (9.3%)	1,643 (19.9%)	262 (3.2%)	5 (0.1%)	66 (0.8%)
pk2030	4,009	1,360 (33.9%)	1,497 (37.3%)	336 (8.4%)	713 (17.8%)	73 (1.8%)	<5 (0.1%)	26 (0.7%)
q2040	8,013	2,641 (33.0%)	3,047 (38.0%)	723 (9.0%)	1,370 (17.1%)	165 (2.1%)	<5 (0.0%)	65 (0.8%)

^a^ Details of the other types of accommodation (e.g. caravan, boat) are available as text.

In Table 3 and Table 4 the lowest floors of the accommodation and the number of rooms used for living and sleeping are summarised. The move to lower floors over time is likely a reflection of families with young children applying for and being moved to a council house or being able to afford a home with a garden for a growing family. There are currently 65 old style (council owned) tower blocks in Bristol (11+ stories). These are situated in more deprived areas: Ashton Gate 3, Lawrence Hill 2, Barton Hill 8, Hartcliffe 8, Brislington 2, Bedminster 8, Redfield 1, Withywood 3, Jacob’s Wells 1, Henbury 3, Easton 3, Shirehampton 3, St Judes 2, Kingsdown 6, St George 2, Redcliffe 8, Lawrence Weston 2. Over the last 20 years or so the gradual redevelopment of the former industrial zone around the harbour, the Avon and Frome rivers has resulted in many new luxury flat developments but these are rarely higher than 7/8 floors and not aimed at the social housing sector. However, planning permission has been given to build two blocks of 20+ stories and others are in discussion ( Bristol Campaign against Tower Blocks, 2022).

**Table 3.** The range in lowest levels of living accommodation (the median at all time points was the ground-level floor).

Variable	Time point	N	Range
a009	Pregnancy	13,251	Basement – 16th
f307	8m	10,785	Basement – 19th
g355	21m	10,029	Basement – 23rd
h265	33m	9,402	Basement – 29th
k5040	5y	8,753	Basement - 15th
m2040	7y	8,176	Basement - 9th
pk2040	7y	3,990	Basement – 9th
q2050	10y	7,952	Basement – 10th

**Table 4.** Numbers of rooms in the home used for living or sleeping. (0 will include bedsits, caravans etc)

Variable	Time point	No. rooms	N	Range
a043	Pregnancy	No. living rooms excluding kitchen	13,291	0–11
a044	Pregnancy	No. bedrooms	13,253	0–9
a045	Pregnancy	Total rooms	13,184	0–18
f343	8m	No. excluding kitchen	11,091	0–12
f344	8m	No. bedrooms	11,054	0–9
f345	8m	Total rooms	11,034	0–19
g413	21m	No. excluding kitchen	10,006	0–17
h293	33m	No. excluding kitchen	9,501	0–18
h294	33m	Total rooms	9,501	1–19
k5070	5y	No. excluding kitchen	8,874	0–18
m2070	7y	No. excluding kitchen	8,181	1–19
pk2070	7y	No. excluding kitchen	3,958	1–30
q2080	10y	No. excluding kitchen	7,616	1–23

Features of the home that were recorded include the type of kitchen (and whether it is large enough for the family to sit and eat), whether there is an indoor flushing toilet, a garden or a balcony. For each of these features, the mother reported whether or not the family has sole use or whether it was shared with others ( Table 5a– Table 5e). It should be noted that the details of whether there is a kitchen without room to sit and eat are not always consistent with the details of the presence of a kitchen with such facilities ( Table 5a and Table 5b); this is sometimes due to a failure to respond to the second question having answered the first but may also indicate that there is more than one kitchen in some homes.

**Table 5a.** Presence of a separate kitchen large enough for the family to sit and eat.

Variable	Time point	N	% sole use	% shared	% do not have
a040	pregnancy	13,346	47.0	2.7	50.3
f340	8m	11,121	48.4	1.1	50.5
g410	21m	9,957	52.8	0.8	46.4
h290	33m	9,571	54.0	0.5	45.5
k5060	5y	8,704	61.5	0.5	38.0
m2060	7y	8,044	63.8	0.4	35.8
pk2060	7y	3,895	64.8	0.5	34.7
q2070	10y	7,760	66.9	0.4	32.7

* Maternal reports in black, mother’s partner reports in red

**Table 5b.** Presence of a separate smaller kitchen in which there is insufficient space for the family to sit and eat.

Variable	Time point	N	% sole Use	% shared	% do not have
a041	pregnancy	13,328	54.6	2.8	40.8
f341	8m	11,09	61.8	1.1	37.1
g411	21m	8.404	72.3	0.8	26.9
h291	33m	9,571	56.7	0.6	42.7
k5061	5y	7,485	70.8	0.6	28.6
m2061	7y	6,575	68.0	0.6	31.4
pk2061	7y	3,299	65.8	0.5	33.7
q2071	10y	6,050	68.4	0.4	31.2

**Table 5c.** Presence of an indoor flushing toilet.

Variable	Time point	N	% sole use	% shared	% None
a042	pregnancy	12,765	92.7	5.2	2.1
f342	8m	10,869	95.3	2.1	2.6
g412	21m	10,062	97.4	1.2	1.4
h292	33m	9,355	97.4	1.0	1.6
k5062	5y	8,859	98.9	0.7	0.4
m2062	7y	8,133	98.9	0.5	0.5
pk2062	7y	3,935	98.5	0.7	0.8
q2072	10y	7,834	98.8	0.6	0.6

**Table 5d.** Presence of a garden or yard.

Variable	Time point	N	% sole use	% shared	% None
a049	pregnancy	13,256	82.6	9.9	7.5
f353	8m	11,139	87.6	6.2	6.2
g418	21m	10,192	90.8	4.4	4.8
h298	33m	9,560	92.9	4.0	3.1
k5083	5y	8,884	95.7	2.3	2.0
m2083	7y	8,269	97.2	1.9	0.9
pk2083	7y	4,015	97.8	1.6	0.6
q2093	10y	8,005	97.9	1.4	0.7

**Table 5e.** Presence of a balcony.

Variable	Time point	N	% sole use	% shared	% none
a050	pregnancy	13,432	6.5	1.0	92.5
f350	8m	11,139	6.7	0.7	92.6
g419	21m	10,058	5.2	0.5	94.3
H299	33m	9,224	6.2	0.6	93.2
k5084	5y	8,560	6.1	0.4	93.4
m2084	7y	8,026	5.7	0.3	94.0
pk2084	7y	3,855	7.8	0.2	92.0
q2094	10y	7,726	5.9	0.1	93.9

### Financial aspects

Housing tenure in the U.K. can be complex, but is a good marker of socio-economic status. It distinguishes between homes that were being bought (mortgaged); were owned outright; rented from the Council or rented from a non-profit housing association (social housing); or rented from a private landlord, the latter can be furnished or unfurnished accommodation ( Table 6). As previously mentioned, the Housing Act of 1980 allowed long-standing council tenants to purchase their home far below market value and about 2% of ALSPAC respondents indicated that they had done this. This also gave them the option to sell that home in the future at current market value.

**Table 6.** Housing tenure: Numbers (%) in each category of housing.

Variable	N	Mort	BBC	OO	RC	PRF	PRUF	HAR	Other
a006	13,335	9,469 * (71.0)	-	288 (2.2)	1,918 (14.4)	575 (4.3)	395 (3.0)	220 (1.7)	470 (3.5)
f304	11,091	8,299 * (74.8)	-	246 (2.2)	1,485 (13.4)	249 (2.3)	354 (3.2)	180 (1.6)	278 (2.5)
g352	10,204	7,691 (75.4)	209 (2.1)	96 (0.9)	1,278 (12.5)	195 (1.9)	340 (3.3)	180 (1.8)	215 (2.1)
h262	9,579	7,290 (76.1)	220 (2.3)	117 (1.2)	1,115 (11.6)	148 (1.5)	297 (3.1)	196 (2.1)	196 (2.1)
k5010	8,898	6,877 (77.3)	74 (0.8)	254 (2.9)	960 (10.8)	105 (1.2)	265 (3.0)	217 (2.4)	146 (1.6)
m2010	8,275	6,535 (79.0)	81 (1.0)	334 (4.0)	714 (8.6)	61 (0.7)	230 (2.8)	180 (2.2)	140 (1.7)
pk2040	4018	3426 (85.3)	33 (0.8)	168 (4.2)	170 (4.2)	28 (0.7)	84 (2.1)	37 (0.9)	72 (1.8)
q2010	8,031	6,280 (78.2)	127 (1.6)	527 (6.6)	548 (6.8)	33 (0.4)	209 (2.6)	197 (2.4)	110 (1.4)
t1010	4,154	2,770 (66.7)	12 (0.3)	953 (22.9)	120 (2.9)	20 (0.5)	134 (3.2)	89 (2.1)	56 (1.4)

* Mortgaged included owned outright; Mort= mortgaged; BBC = being bought from the Council; OO = Owned outright; RC = Rented from the Council; PRF = private rented, furnished; PRUF = private rented unfurnished; G = Housing Association rented; Other = eg. Boat, caravan (details available as text)

All homes are expected to pay a Council Tax, the amount (known as the tax band) being related to the size, layout, character, location and value of the home. Bands are based on the rateable value of the property as of 1 ^st^ April 1991 and rise on average about 2–3% per annum. The current (2022) tax amounts per annum for Bristol City Council range from the lowest band, A (£1,487) to the highest H/I (£4,461). Other councils covering the ALSPAC area are South Gloucestershire and North Somerset, which have slightly lower rates in 2022 than Bristol. This tax covers the costs of the police, education, social care and facilities such as waste and recycling collection, local libraries and upkeep of public spaces, etc. The Council Tax bands of the homes in which the mothers were living when the study children were aged 10 and 18 are shown in Table 7.

**Table 7.** The Council Tax band of the study participants’ homes (A is the lowest; H/I the highest).

Variable	N	A	B *	C *	D	E	F	G	H/I
q2020	5,178	233 (4.5%)	1,319 (25.5%)	1,241 (24.0%)	1,169 (22.6%)	607 (11.7%)	335 (6.5%)	230 (4.4%)	44 (0.8%)
t1013	2,750	83 (3.0%)	550 (20.0%)	652 (23.7%)	713 (25.9%)	358 (13.0%)	216 (7.9%)	146 (5.3%)	32 (1.2%)

*The median Council Tax band in Bristol is B, and C in S Glos and N Somerset.

Information was collected on whether the parents reported having difficulty in paying for their accommodation. Table 8 shows that the proportion stating they had difficulties reduced over time.

**Table 8.** How difficult it is to pay the rent or mortgage.

Variable Name	N	Time point	Very difficult	Fairly difficult	Some difficulty	Not difficult	Paid by Social Security #
c523	11,999	Pregnant	4.6%	7.7%	17.9%	69.7%	NA
f803	11,114	8 m	4.8%	8.4%	19.3%	67.6%	NA
pd683	7,052		5.5%	9.3%	22.9%	62.3%	NA
g838	10,065	21m	3.3%	7.2%	16.6%	64.7%	8.2%
pe463	6,066	21m	3.4%	6.9%	19.1%	66.3%	4.3%
k6203	8,772	5 years	1.6%	4.2%	13.8%	72.0%	8.5%
r9003	7,426	11 years	1.1%	2.6%	10.3%	76.1% *	NA
pp9003	3,558	11 years	0.7%	2.1%	10.6%	81.1%	5.5%

*a further 10.1% stated that they were not responsible for paying; NA = not asked # Monetary assistance from the state for people with an inadequate or no income.

### Stability of the home

Many of the mothers moved home before and during the child’s life: three-quarters had moved in the 5 years prior to the pregnancy, and subsequently over 10% had moved during each of the first 4 years of the child’s life ( Table 9). In parallel there were small numbers of mothers and their partners who had been made homeless during the pregnancy and subsequently, this reduced over time ( Table 10). Reported homelessness is likely to mean the family unit may have sought emergency accommodation via the City Council and/or resided in temporary accommodation, such as a ‘Bed and Breakfast’ or hotel or with family and friends.

**Table 9.** Moving home.

Moved during period	Variable name	Proportion (N) moved
From 5 years before pregnancy	a005	75.9% (9917)
	pa005	74.6% (6239)
1 ^st^ half of pregnancy	b591	10.2% (1214)
	pb181a	10.5% (1040)
1 ^st^ 7m of pregnancy	c470	13.8% (1654)
Mid-pregnancy to 4wk PP	e421	12.2 % (1418)
Mid-pregnancy to 8 wks PP	pc221a	11.7% (972)
In 1 ^st^ 8 months PP	f302	14.1% (1523)
	pd241a	10.8% (758)
8 –21m	g321a	16.7% (1718)
	pe321a	15.1% (927)
18 – 33m	h231	19.9% (1903)
	pf5021	18.3% (998)
30 – 47m	j321	19.6% (1878)
	pg3021	17.7% (900)
4 – 5y	k4021a	13.1% (1170)
	ph4021	12.8% (575)
5 – 6y	l4021	12.7% (1079)
	pj4021	12.2% (542)
6 – 9y	p4021	24.2% (1896)
	pm2021	23.0% (831)
At 10y		
9 – 11y	r5021	176.09% (12746)
	pp5021	16.0% (578)
17 – 18y	t3320	4.4% (181)

^a^No. of moves in period of time; ^b^Age or date at last move PP = postpartum

**Table 10.** Made homeless.

Became homeless during period	Variable name	Proportion (n) homeless
1 ^st^ half of pregnancy	b593	1.7% (205)
	pb183	1.2% (121)
1 ^st^ 7m of pregnancy	c472	2.3% (268)
Mid-pregnancy to 4wks PP	e423	1.3% (148)
Mid pregnancy to 8wks PP	pc223	1.1% (88)
First 8 months PP	f243a	2.2% (125)
	pd243	0.7% (52)
8 –21m	g323a	1.0% (105)
	pe323	0.5% (30)
30 – 47 m	j323	0.8% (78)
	pf5023	0.7% (39)
4 – 5y	k4023a	0.5% (47)
	pg3023	0.5% (25)
5 – 6y	l4023	0.6% (49)
	ph4023	0.3% (15)
6 – 9y	p4023	0.8% (62)
	pj4023	0.4% (16)
9 – 11y	r5023	0.5% (35)
	pm2023	0.3% (12)
17 – 18y	t3322	0.1% (5)

PP = postpartum

Not all families were living in their own home – during pregnancy 6% were living in their parents’ home, but this reduced to around 1% by the time the children were aged over 2 years, and less still as time went on ( Table 11).

**Table 11.** Whether the mother lives in her own home, with her parents or with others.

Variable name	Time point	N	Own Home	Partner’s Home	Parent’s Home	Partner’s Parents Home	Other
a007	Pregnancy	13,232	11,747 (88.8%)	-	801 (6.1%)	-	684 (5.2%)
f305	8m	11,049	10,432 (94.4%)	-	355 (3.2%)	-	262 (2.4%)
g353	21m	10,146	9,698 (95.6%)	119 (1.2%)	151 (1.5%)	32 (0.3%)	146 (1.4%)
h263	33m	9,489	9,116 (96.1%)	100 (1.1%)	103 (1.1%)	20 (0.2%)	147 (1.6%)
k5020	5y	8,572	8,572 (97.2%)	66 (0.8%)	64 (0.7%)	18 (0.2%)	102 (1.2%)
m2020	7y	8,243	7,991 (96.9%)	99 (1.2%)	51 (0.6%)	14 (0.2%)	88 (1.1%)
pk2020	7y	4002	3856 (96.4%)	76 (1.9%)	12 (0.3%)	7 (0.2%)	51 (1.3%)
q2030	10y	7,979	7,793 (97.7%)	58 (0.7%)	33 (0.4%)	6 (0.1%)	89 (1.1%)

### Strengths and limitations

The strength of this data is that the ALSPAC dataset is large with over 14,000 mothers initially enrolled in the study ( Boyd *et al.,* 2013) and the ongoing support and commitment from the study families. The only inclusion requirements at enrolment were the geographical location of residence and the expected date of delivery. The participants recruited to the study were broadly representative of the general population of new parents resident in the area at the time in terms of sex, ethnicity and socio-economic status ( Fraser *et al*., 2013).

Further strengths include the breadth and frequency of data collection with availability of repeat measures (e.g. of health and mental health, child development); an extensive biobank and the breadth of genetic sampling and proteomics data; geocoded residence and school history data from pregnancy onwards. In addition, some data is available on the parents and grandparents of the original parent cohort mainly in regard to life events, place of birth and household moves, demographics and smoking history. Children born to the offspring cohort are also being followed-up. This makes the data very flexible and relatable to intergenerational aspects of the family’s life.

A limitation of this study is the lack of diversity, because at the time of enrolment, the county of Avon was mainly Caucasian, therefore there were too few non-white participants (<6% in all) to allow for detailed analysis by ethnic background. Increasing attrition means that the cohort has an over-representation of the more affluent, those with higher educational attainment and females. ALSPAC invests considerable resources into collecting data from health and administrative records to track participants, and participation initiatives aimed at increasing response rates.

## Ethical approval and consent

Prior to commencement of the study, approval was sought from the ALSPAC Ethics and Law Committee and the Local Research Ethics Committees. Consent for the use of data collected via questionnaires and clinics was implied from participants following the recommendations of the ALSPAC Ethics and Law Committee at the time. Questionnaires were completed in the participants own home and the return of the questionnaires was taken as continued consent for their data to be included in the study ( Birmingham, 2018). Full details of the approvals obtained are available from the study website. Study members have the right to withdraw their consent for elements of the study or from the study entirely at any time.

## Figures and Tables

**Appendix 1.** Summaries of a sample of ALSPAC publications using external housing environment data.

Author	Brief summary
Boyd *et al.*, 2019	Describes ALSPAC data concerning the physical and social environment including spatial and temporal information collected on the three generations (parents (G0), offspring (G1), grandchildren (G2)) on: (a)Area-level built and social characteristics (e.g. neighbourhood crime rates, density and location of fast-food retailers); (b)Exposure measurements (such as temperature and air pollution concentrations including nitrogen dioxide, radiation and electrical magnetic fields); (c)Respondent-reported data relating directly to places and spaces (e.g. homes and schools) that they inhabit (for example, neighbourhood safety, traffic density, the indoor environment of their home); (d)Direct measurements on the participants (e.g. blood lead, cadmium and total mercury concentrations and other heavy metals and VOCs); (e)Location information required to link these diverse data. *This paper and its supplementary material list many publications mainly using the internal housing environment*.
Baker *et al.*, 1998	Examined the effect of deprivation, crowding, maternal smoking and breast feeding on infant morbidity from wheeze and diarrhoea in the first 6 months of life. Deprivation was associated with heightened morbidity from wheeze and diarrhoea, but results suggested that breast feeding was protective was particularly associated with reduced severity and duration.
Sidebotham *et al.*, 2002	Four indicators of deprivation all showed significant relationships with the child being on the at-risk register. Adjusted odds ratios were 2.33 for paternal unemployment; 7.65 for council housing; 2.16 for overcrowding; and 2.33 for car ownership. There was a strong relationship between the number of indicators of deprivation and the risk of maltreatment. In a second model, maternal unemployment, high mobility (> 3 house moves in the previous 5 years) and a poor social network were also significant with odds ratios of 2.82, 2.81, and 3.09, respectively.
Baker & Henderson, 1999	63.4% (1239) of an ALSPAC sub-sample lived in owner occupied/mortgaged accommodation (relatively affluent) and 36.6% (715) lived in council house/rented accommodation (relatively deprived). Wheeze was significantly more likely for infants living in council house/rented accommodation (p < 0.0001), their mothers (p < 0.001) and their fathers (p < 0.01).
Chan *et al.*, 2021	Emotional and physical IPV and financial adversity independently and jointly increased the risk of incident homelessness. The effects of emotional and physical IPV are comparable to or greater than the risk of financial adversity.
Taylor *et al.*, 2013	The strongest categorical predictor of blood lead levels (BLL) was having a coal fire (p = 0.008), followed by highest education attainment (p,0.001), haemoglobin level (p = 0.014), alcohol consumption (p = 0.001), age (p = 0.006), cigarette smoking (p = 0.001) and coffee consumption (p,0.001). Dietary calcium intake was protective against high BLL (p = 0.041). Neighbourhood quality index also assessed.
Fuertes *et al.*, 2020	This study provided some evidence that children living in more vegetated places or live in close proximity to green spaces have better lung function up to 24 years of age. It used repeated greenspace and lung function data at 8, 15 and 24 years.
Beale *et al.*, 2006	Tested if CTVB (Council Tax Valuation Band) of maternal home address at the time of delivery predicted breast- feeding and personal and socio-economic attributes of the mothers. Random selection of 1390 mothers divided equally between all CTVBs during pregnancy with outcomes at 8 weeks postpartum (attitudes to breast-feeding prior to delivery; breast-feeding intention and uptake; demographic and socio-economic attributes of the mothers). CTVB predicted only one attitude: maternal convenience of bottle-feeding. Only a third of infants in the lowest CTVB band (A) were still fully breast fed at 4 weeks, whereas 57% of those living in the highest bands CTVB (E+) were fully breast fed. CTVB was strongly associated with maternal social class, home conditions, parental educational attainment, family income and smoking habit.
Bowen *et al.*, 2005	Looked at incidence of domestic violence at 5 timepoints (during pregnancy to 33 months post-partum). Found inadequate housing (crowding index/periods of homelessness); availability of basic amenities in the home (no hot water or no indoor toilet, bath or shower or no kitchen); housing defects/infestation and financial difficulties were all significant at P<0.001 for emotional or physical abuse (or both).

## Data Availability

ALSPAC data access is through a system of managed open access. The steps below highlight how to apply for access to the data included in this paper and all other ALSPAC data. Please read the ALSPAC access policy which describes the process of accessing the data and biological samples in detail, and outlines the costs associated with doing so. You may also find it useful to browse our fully searchable research proposals database, which lists all research projects that have been approved since April 2011. Please submit your research proposal ( https://proposals.epi.bristol.ac.uk/) for consideration by the ALSPAC Executive Committee using the online process. You will receive a response within 10 working days to advise you whether your proposal has been approved. If you have any questions about accessing data, please email: alspac-data@bristol.ac.uk (data) or bbl-info@bristol.ac.uk (samples). Genette Ellis – Formal analysis, Investigation, Project administration, Writing – Original draft presentation, Writing - Review and editing, Validation, Visualisation. Yasmin Iles-Caven - Funding acquisition, Project administration, Writing – Review and editing. Jean Golding – Conceptualization, Funding acquisition, Investigation, Project administration, Resources, Supervision, Writing - Review and editing. Kate Northstone – Funding acquisition, Writing - Review and editing.
