# Supplementary material for: The external housing environment of participants in the Avon Longitudinal Study of Parents and Children (ALSPAC): a resource for studies of influences on health
Source: Wellcome Open Res. 2022 Dec 21;7:232. Originally published 2022 Sep 12. [Version 2] doi: 10.12688/wellcomeopenres.17996.2 (PMC10624949; doi:10.12688/wellcomeopenres.17996.2)
Supplement: Peer review history

# Reviewer report: Reviewer response for version 2

**DOI:** 10.21956/wellcomeopenres.20699.r72314

**Published:** 2024-01-26

**Authors:** Marlee Bower

**Affiliations:** 1 The University of Sydney, Sydney, Australia

This article describes a dataset of the housing conditions of 13,000 women during and over the 18 years after pregnancy, using the Avon Longitudinal Study of Parents and Children. The dataset refers to people residing in Bristol area.  I think the dataset looks really exciting and provides a lot of fascinating covariates which will be invaluable for housing researchers to access and use.

 I commend the authors for careful consideration of past reviewer comments, particularly around clarification around living/sleeping conditions, homelessness etc.

 In saying that, I think that there are still certain improvements that could be made to the manuscript in order to improve accessibility and readability. Firstly, I know that this has been covered by several previous reviewers, but I think the authors need to remove the over-detailed information about the history of the Bristol housing context. The full second paragraph starting "There is evidence that Bristol..." should be removed. Also the sixth paragraph starting "By far, the greatest impact on house building in the Georgian period..." should be removed. Neither provides useful context to the housing data and actually is quite hard to follow. The rest of the introduction could also be tightened to remove extraneous historical detail, keeping text limited to how the historical context shaped the physical structure of housing or impacted the nature of housing tenure.

 More evidence should be provided for the variables included in the database, especially when they are purported to also act as a proxy to other broader social indicators, e.g. "Housing tenture in the U.K. can be complex, but it is a good marker of socio-economic status." (p9) or "The move to lower floors over time is likely a reflection of families with young children applying for and being moved to a council house or being able to afford a home with a garden for a growing family" (p9). 

Are sufficient details of methods and materials provided to allow replication by others?

Yes

Is the rationale for creating the dataset(s) clearly described?

Yes

Are the datasets clearly presented in a useable and accessible format?

Yes

Are the protocols appropriate and is the work technically sound?

Yes

Reviewer Expertise:

Housing and its link to mental health and social wellbeing

I confirm that I have read this submission and believe that I have an appropriate level of expertise to confirm that it is of an acceptable scientific standard.

# Reviewer report: Reviewer response for version 2

**DOI:** 10.21956/wellcomeopenres.20699.r72307

**Published:** 2024-01-26

**Authors:** Marcella Ucci

**Affiliations:** 1 University College London (UCL), London, UK

Overall the protocol clearly articulates key details of the dataset and its value. I provide below some suggestions which the authors may wish to consider: 

1. The section with the historical overview of Bristol and the housing stock is interesting. However it would be useful, if possible, to also provide some relevant details on housing type/stock/tenure from the Census (the latest and the past one?). This would give a clearer understanding of how the area which has been described in a historical sense may relate to other areas, by providing means for comparison via Census information.  
2. Accordingly, a more specific discussion of some of the key housing variables could be provided, in relation to other metrics. For example, how are metrics such as the number of room variable related to similar metrics in the Census, or the English Housing Survey or the Understanding Society Dataset? i.e. to what extent are they similar variables (e.g. is it number of rooms or rooms used for sleeping). 
3. Although I understand that data dictionary etc. is available elsewhere and links were provided, I did find it rather time-consuming to look for which questions/answers were asked for the housing variables. Could some further information be provided to assist the reader with this task, perhaps more specific links to sections of the questionnaires with the housing variables, or include in an appendix the name of the variables so one might search them more easily in the various other resources already available online? 
4. It is noted that this version has a title which refers to 'external housing environment': I am not sure how useful this is. How is tenure or number of rooms relevant to 'external housing environment'? Not sure this reference to 'external' helps explain the focus of this aspect of the dataset/protocol. 

I trust these comments are useful and help complement an otherwise excellent submission.

Are sufficient details of methods and materials provided to allow replication by others?

Partly

Is the rationale for creating the dataset(s) clearly described?

Yes

Are the datasets clearly presented in a useable and accessible format?

Partly

Are the protocols appropriate and is the work technically sound?

Yes

Reviewer Expertise:

Housing and health, environmental design and engineering, buildings and health/wellbeing

I confirm that I have read this submission and believe that I have an appropriate level of expertise to confirm that it is of an acceptable scientific standard.

# Reviewer report: Reviewer response for version 2

**DOI:** 10.21956/wellcomeopenres.20699.r68441

**Published:** 2023-11-03

**Authors:** Alexandra Farrow

**Affiliations:** 1 School of Health and Lifesciences, Brunel University London, London, UK

This scientifically sound paper provides a unique historical record of the external environment of the participants of the 13,000 participants of the ALSPAC cohort. As such it will be an asset to a varied academic audience. The social aspects that have been recorded as the study followed the participants is not likely to be repeated.

Are sufficient details of methods and materials provided to allow replication by others?

Yes

Is the rationale for creating the dataset(s) clearly described?

Yes

Are the datasets clearly presented in a useable and accessible format?

Yes

Are the protocols appropriate and is the work technically sound?

Yes

Reviewer Expertise:

Epidemiology and environmental exposures

I confirm that I have read this submission and believe that I have an appropriate level of expertise to confirm that it is of an acceptable scientific standard.

# Reviewer report: Reviewer response for version 1

**DOI:** 10.21956/wellcomeopenres.19953.r52709

**Published:** 2022-11-11

**Authors:** Rebecca Bentley

**Affiliations:** 1 Centre for Health Policy, Melbourne School of Population and Global Health, University of Melbourne, Melbourne, Vic, Australia

This data note provides information on housing data collected as part of the Avon Longitudinal Study of Parents and Children from 1991.

 While the title of the paper suggests a focus on home environments, this data note focusses specifically on tenure, type of home, size and overcrowding, presence of amenities and frequency of moves. There is a second data note planned that will cover internal housing conditions (which some researchers might equate more closely with home environments).

 The section entitled ‘historical background and manmade environment’ contains historical detail on the geographic area that dates to the stone age. While interesting, much of this text is not relevant to the housing-health relationships covered from 1991 on. This section, and some of the text of housing stock, could be much more succinctly summarised. Of use to readers most is information pertaining to noise and air pollution from motorways in the area.

 The data note could be made more useful to housing and health researchers with two additional sets of information:

1. More on the housing and health relationships that are explorable using these data (flagging any sources of bias). The paper broadly describes physical and mental health and childhood development as health outcomes. It would be useful to know how the health information maps to this housing information more specifically (e.g., asthma, allergies etc.) and what research has already been undertaken using these data. I note also that all measures are self-reported and issues relating to misclassification bias could also be addressed in this note. 
2. An overarching framework for conceptualising housing as an exposure. For example, using the concepts of housing affordability, security and suitability to categorise and group available measures (e.g., see Mansour et al 2022) ^1^. These interrelated terms are useful for organising the many dimensions of measurement available on housing and an exposure that affects health. This might also offer a way to tie data notes one and two together (given that housing affordability, for example, is likely to be associated with housing condition). Authors might also consider these measures within a life course framework. 

Are sufficient details of methods and materials provided to allow replication by others?

Yes

Is the rationale for creating the dataset(s) clearly described?

Yes

Are the datasets clearly presented in a useable and accessible format?

Yes

Are the protocols appropriate and is the work technically sound?

Partly

Reviewer Expertise:

social epidemiology

I confirm that I have read this submission and believe that I have an appropriate level of expertise to confirm that it is of an acceptable scientific standard, however I have significant reservations, as outlined above.

## References

[1] : Housing and health: an updated glossary. *J Epidemiol Community Health* .76(9) : 10.1136/jech-2022-219085 833-838 10.1136/jech-2022-219085 35760516

# Response

**Published:** 2022-12-01

**Authors:** Yasmin Iles-Caven

**Affiliations:** University of Bristol, UK

**We have added the word ‘external’ to the title and hope this clarifies the content.**

 The section entitled ‘historical background and manmade environment’ contains historical detail on the geographic area that dates to the stone age. While interesting, much of this text is not relevant to the housing-health relationships covered from 1991 on. This section, and some of the text of housing stock, could be much more succinctly summarised. Of use to readers most is information pertaining to noise and air pollution from motorways in the area.

**As in our response to Reviewer** 1, we gave such historical detail to aid international researchers who may not be familiar with the evolution of Bristol and the Avon area and it’s effect on housing and open spaces for example. The detail around the Bristol Blitz in WW2 is pertinent to this cohort where we have information on housing type from the grandparents to the index offspring and now their own children.

 The data note could be made more useful to housing and health researchers with two additional sets of information:

1. More on the housing and health relationships that are explorable using these data (flagging any sources of bias). The paper broadly describes physical and mental health and childhood development as health outcomes. It would be useful to know how the health information maps to this housing information more specifically (e.g., asthma, allergies etc.) and what research has already been undertaken using these data. I note also that all measures are self-reported and issues relating to misclassification bias could also be addressed in this note. 

**Response**: We have added text along the lines of the following: There are 2000+ papers published using ALSPAC data. The authors have selected a few papers that specifically mention ‘housing’ in relation to health outcomes and we have put these in an additional table (Appendix 1).

 In regard to misclassification bias, whilst this is possible in self-reported data, tenure and type of home is unlikely to be erroneous. The address histories have been geocoded and enables linkage to the Index of Multiple Deprivation (IMD) for which we have derived variables, and also the wider environment such as proximity to main roads, green spaces and power lines. Geocoding could also clarify any misclassification errors.  For those wishing to research the broader external environment it is worth reading this paper in conjunction with Boyd et al., 2019.

1. An overarching framework for conceptualising housing as an exposure. For example, using the concepts of housing affordability, security and suitability to categorise and group available measures (e.g., see Mansour et al 2022) ^1^ . These interrelated terms are useful for organising the many dimensions of measurement available on housing and an exposure that affects health. This might also offer a way to tie data notes one and two together (given that housing affordability, for example, is likely to be associated with housing condition). Authors might also consider these measures within a life course framework. 

**Response:** It is feasible to consider the housing measures in this paper within the life course of both the parent and offspring cohorts.  We agree that an overarching framework to conceptualise housing as an exposure would be useful, but is without the remit of this purely descriptive paper.

# Reviewer report: Reviewer response for version 1

**DOI:** 10.21956/wellcomeopenres.19953.r52378

**Published:** 2022-10-27

**Authors:** Anna L. Hansell

**Affiliations:** 1 Centre for Environmental Health and Sustainability, University of Leicester, Leicester, UK

The article describes data on housing environment of individuals in the ALSPAC study. The title states the housing environment data provide “a resource for studies of influences on health”. 

 The paper provides an interesting description of the variables held, but a major limitation is that it currently doesn’t give any examples of how they have been used in health studies or how the authors consider they could be used in health studies in the future. The housing variables are considered separately from other measures such as deprivation/education/household income, or indoor environment (the latter of which I understand is the subject of another paper). 

 The introduction ‘Historical background and the manmade environment’ going back to the Stone Age is interesting but didn’t seem particularly relevant to future health studies – particularly as this would potentially impact all individuals in the study. It doesn’t help with use of the dataset (except perhaps the last paragraph about the motorways).  Similar issues apply to the paragraph on bombing during the Second World War. Additionally, it mainly relates to Bristol, while the paper states many of the families live in surrounding areas. I would suggest this information is shortened.

 Standard of Housing – page 4.  This paragraph has two possibly contradictory statements. Firstly “Older buildings are more at risk of poor standards” and secondly “in Bristol, children living in older, more affluent areas of the city were less likely to experience poverty”. Please could wording be looked at.

 Figure 4 – typical Bristol housing.  It’s unclear if this actually relates to housing stock lived in by participants. E.g. are there any families living in prefabs?

 Results – there will be drop out from the study over time and this is usually in families that are more disadvantaged, so you end up with a wealthier cohort than the one you started with. Therefore housing types will differ over time due to selection bias. Has this been looked at?  If not, then at least a table on SES of those leaving the study to illustrate the selection bias would be useful. How many of these are moving out of study area and how many are lost to follow-up?

 There is some variability with percentages in a lot of the tables and I wondered if this was related to response rate for the questionnaire so there is some chance variability (as well as bias). Is it relevant to add this here or at least to Table 1.

 Table 2 and subsequent tables. Table 2 in particular didn’t have the key that the ‘a’ in front of the variable means it relates to the ‘A’ questionnaire – it took me a while to work this out. Please could time-point be added or a footnote be added to clarify this.

 Table 3 – is the move to lower floors a reflection of a city policy to knock down tower blocks or is it that families with small children are moving?

 Table 4 – How is it possible to have zero living or sleeping rooms in a house (ranges start from zero)? Please could this be explained. This suggests a house with only toilet and/or kitchen.  It would also be useful to see the median here.

 Table 5c. The last row adds up to 99.3%, while the rest of the rows add up to 100%. Please could this be checked. If due to missing data, a footnote would be useful.

 Table 6 Housing tenure.  English housing tenure is a complicated area and something it would be worth explaining more in the introduction or methods as to why these categories were chosen and are meaningful. E.g. being bought from council – will be council housing but this is sometimes then sold on.  It also relates more strongly to certain political eras. Private rented had fewer regulations in the 1980s but is better regulated now with insulation, safety certificates etc.   Also, it’s unclear whether housing tenure is a marker for SES or environmental conditions or social conditions/neighbourhood or all of these.  How does this correlate with other measures of SES? Have these analyses been conducted?

 Table 9. Moving home – how does this compare with other cohorts e.g. Born in Bradford?  Is there a cohort effect or a geographical effect? Also the Longitudinal Survey looks at how far people move from the original home – have any such analyses been conducted for ALSPAC?  This comparison information would be useful.

 Table 10. Please could the definition of homeless used be included.

Are sufficient details of methods and materials provided to allow replication by others?

Yes

Is the rationale for creating the dataset(s) clearly described?

Yes

Are the datasets clearly presented in a useable and accessible format?

Yes

Are the protocols appropriate and is the work technically sound?

Partly

Reviewer Expertise:

Environmental epidemiology

I confirm that I have read this submission and believe that I have an appropriate level of expertise to confirm that it is of an acceptable scientific standard, however I have significant reservations, as outlined above.

# Response

**Published:** 2022-12-01

**Authors:** Yasmin Iles-Caven

**Affiliations:** University of Bristol, UK

**Thank you for your thorough and considered review**.

 You said: The introduction ‘Historical background and the manmade environment’ going back to the Stone Age is interesting but didn’t seem particularly relevant to future health studies – particularly as this would potentially impact all individuals in the study. It doesn’t help with use of the dataset (except perhaps the last paragraph about the motorways).  Similar issues apply to the paragraph on bombing during the Second World War. Additionally, it mainly relates to Bristol, while the paper states many of the families live in surrounding areas. I would suggest this information is shortened.

**Respons**e: We have altered the title to make it clear we are describing the external housing environment.

 We have changed ‘Many ALSPAC families’ to ‘About 25% ...’. We have added text that the small towns and villages often reflect similar growth to Bristol (radiating out from the historical centre) and expanded on the growth and reasons for social housing. Our main reason for including the historical background was for an international audience, to explain the extent of ancient to post-war modern buildings radiating out from what was old Bristol centered on the Castle and old walled city.  In addition, data has been collected from the parents on the type of housing in which they lived as children and so we have multigenerational housing encompassing the index offspring back to their grandparents (many of whom lived through the Second World War).

 You said: Standard of Housing – page 4.  This paragraph has two possibly contradictory statements. Firstly “Older buildings are more at risk of poor standards” and secondly “in Bristol, children living in older, more affluent areas of the city were less likely to experience poverty”. Please could wording be looked at. 

**Response**: We have expanded on this to make it clearer.

 You said: Figure 4 – typical Bristol housing.  It’s unclear if this actually relates to housing stock lived in by participants. E.g. are there any families living in prefabs?

**Response**: We have added to this slightly. There was the option in the questionnaires for ‘Other’ (types of housing) and the text answers included houseboats, prefabs and caravans for example.

 You said: Results – there will be drop out from the study over time and this is usually in families that are more disadvantaged, so you end up with a wealthier cohort than the one you started with. Therefore housing types will differ over time due to selection bias. Has this been looked at? 

**Response**: Whilst this is true of ALSPAC in that participation some 30 years since enrolment has declined and those parents still participating are more likely to have a degree, to be female and more affluent and less likely to have suffered adversity in the index pregnancy or be from a non-white background, it is also true that housing type over time will differ due to selection bias. However, in Britain at least, younger families are more likely to live in cheaper flats/small houses/council or shared accommodation until they can get established on the property ladder and by middle age are more likely to live in a more expensive home in a favourable neighbourhood.  Within the last 20+ years, things have changed considerably. Currently, in the Avon area, monthly rents are often more expensive than a mortgage repayment. The ability of the younger generation to amass a deposit of tens of thousands of pounds means that first time buyers are likely to be much older than they were a generation ago.

 We are not aware of any research specifically looking at housing type over time and potential biases therein.  However, this is eminently feasible in this cohort with the data described in this publication.

 You said: If not, then at least a table on SES of those leaving the study to illustrate the selection bias would be useful. How many of these are moving out of study area and how many are lost to follow-up?

**Response**: Since enrolment, the mothers/families have always been encouraged to continue participating in the study even if they moved out of area (including abroad).  Since 2014 online questionnaires enable contact with participants even more feasible.  For the clinic examinations, ‘out of area’ participants are still invited, and travel/accommodation expenses reimbursed up to a ceiling, including air travel. Even so, ALSPAC goes to considerable lengths to attract the disengaged and track those lost to follow-up.  We have enhanced the Strengths and Limitations section.

 You said: There is some variability with percentages in a lot of the tables and I wondered if this was related to response rate for the questionnaire so there is some chance variability (as well as bias). Is it relevant to add this here or at least to Table 1.

**Response:** We have double-checked all the tables, apart from a few typos, the variability with percentages often 99.9 or 100.1 are due to rounding.

 You said: Table 2 and subsequent tables. Table 2 in particular didn’t have the key that the ‘a’ in front of the variable means it relates to the ‘A’ questionnaire – it took me a while to work this out. Please could time-point be added or a footnote be added to clarify this.

**Response**: We have added a sentence in Table 2’s title as to the variable naming convention and to refer to Table 1 for the timing of the questionnaire.

**You said**: Table 3 – is the move to lower floors a reflection of a city policy to knock down tower blocks. Or is it that families with small children are moving?  

**Response**: The move to lower floors is more a reflection of families with young children applying for and being moved from a flat to a council house. We have added some text about Bristol’s tower blocks.

 You said: Table 4 – How is it possible to have zero living or sleeping rooms in a house (ranges start from zero)? Please could this be explained. This suggests a house with only toilet and/or kitchen.  It would also be useful to see the median here.

**Response**:The range beginning at zero  includes bedsits or studio flats and also caravans and houseboats.

 You said: Table 5c. The last row adds up to 99.3%, while the rest of the rows add up to 100%. Please could this be checked. If due to missing data, a footnote would be useful.

**Response**: This is a typo and has been corrected

 You said: Table 6 Housing tenure.  English housing tenure is a complicated area and something it would be worth explaining more in the introduction or methods as to why these categories were chosen and are meaningful. E.g. being bought from council – will be council housing but this is sometimes then sold on.  It also relates more strongly to certain political eras. Private rented had fewer regulations in the 1980s but is better regulated now with insulation, safety certificates etc.  Also, it’s unclear whether housing tenure is a marker for SES or environmental conditions or social conditions/neighbourhood or all of these.  How does this correlate with other measures of SES? Have these analyses been conducted?  

**Response**: We are unaware of comparative analyses looking at all of these, but tenure certainly has been used as a marker for SES. Please see Appendix 1 for a selection of papers.

 You said: Table 9. Moving home – how does this compare with other cohorts e.g. Born in Bradford?  Is there a cohort effect or a geographical effect? Also the Longitudinal Survey looks at how far people move from the original home – have any such analyses been conducted for ALSPAC?  This comparison information would be useful.

**Response**: The authors are unaware of any comparisons with other cohorts.  The aim of this data note is to describe the data available in ALSPAC and thus enable researchers to undertake comparative research if they wish. 

 You said: Table 10. Please could the definition of homeless used be included. **We have done this.**
